# Maleic Acid as a Co-Former for Pharmaceutically Active GABA Derivatives: Mechanochemistry or Solvent Crystallization?

**DOI:** 10.3390/ma16062242

**Published:** 2023-03-10

**Authors:** Daniel Komisarek, Ebru Taskiran, Vera Vasylyeva

**Affiliations:** Inorganic and Structural Chemistry I, Heinrich-Heine-University, 40225 Düsseldorf, Germany

**Keywords:** solubility, crystal engineering, mechanochemistry, crystal synthesis

## Abstract

In this study, we compare the mechanochemical and classical solvent crystallization methods for forming maleates of GABA and its pharmaceutically active derivatives: Pregabalin, Gabapentin, Phenibut, and Baclofen. Common characterization techniques, like powder and single crystal X-ray diffraction, IR-spectroscopy, differential scanning calorimetry, thermogravimetric analysis and ^1^H-NMR spectroscopy, are used for the evaluation of structural and physicochemical properties. Our work shows that maleate formation is possible with all investigated target compounds. Large increases in solubility can be achieved, especially for Pregabalin, where up to twentyfold higher solubility in its maleate compared to the pure form can be reached. We furthermore compare the mechanochemical and solvent crystallization regarding quickness, reliability of phase production, and overall product quality. A synthetic route is shown to have an impact on certain properties such as melting point or solubility of the same obtained products, e.g., for Gabapentin and Pregabalin, or lead to the formation of hydrates vs. anhydrous forms. For the GABA and Baclofen maleates, the method of crystallization is not important, and similarly, good results can be obtained by either route. In contrast, Phenibut maleate cannot be obtained pure and single-phase by either method. Our work aims to elucidate promising candidates for the multicomponent crystal formation of blockbuster GABA pharmaceuticals and highlight the usefulness of mechanochemical production routes.

## 1. Introduction

In medicinal science, increasing the solubility of active pharmaceutical ingredients (APIs) to improve their bioavailability is a core objective. Several methods have been established to address this concern, including the use of prodrugs [1,2,3,4,5], nanosuspensions, [6,7,8,9] or complexation of APIs [10,11,12,13,14], and modification of the solid API phase itself. The latter approach can be achieved through the formation of API salts, co-crystals, or amorphous systems to enhance the targeted drug’s solubility properties [15,16,17,18,19,20,21,22,23,24,25]. Product solubility is influenced by various thermodynamic and kinetic factors, such as the solubilities of the co-formers, solvent environment, and molecular characteristics like polarization or ionization [17,26,27]. Furthermore, when producing salts or co-crystals for pharmaceutical applications, the counterion or co-former should not show any unwanted or damaging properties. Generally Recognized As Safe (GRAS) compounds, which are approved substances by the U.S. Food and Drug Administration (FDA), are commonly added to API formulations without the need for additional risk assessments [28]. Maleic acid, a simple dicarboxylic acid and a GRAS-list member is a popular choice for salt or co-crystal formation [29,30,31,32,33]. Maleic acids with a pKa_1_ value of 1.74 and pKa_2_ value of 5.81, respectively, make it more likely to form maleate salts instead of co-crystalline compounds due to the larger ΔpKa values between maleic acid and possible co-formers, which shift products in the salt direction on the *salt-cocrystal continuum* [34,35]. The predictability of maleic acid in forming salts with APIs, in conjunction with its low cost, makes it a valuable tool in the pharmaceutical industry, particularly in the formation of the maleates of APIs derived from γ-amino butanoic acid (GABA), in addition to other uses. 

GABA is a non-essential amino acid that regulates sleep, pain, and stress impulses in various life forms [36,37,38,39,40]. Some of the APIs derived from GABA, such as Gabapentin, Pregabalin, Phenibut, and Baclofen, have been found to be effective in treating epilepsy, neuropathic pain, anxiety, and addiction, among other conditions [41,42,43,44,45,46,47,48,49]. A Gabapentin maleate, an (*S*)-Pregabalin maleate hydrate and maleates of racemic and enantiopure Baclofen have been described in the literature [50,51,52,53]. These past works highlight the promising prospect of forming maleates of other related substances as well. GABA, Gabapentin, Pregabalin, Baclofen and Phenibut share similar pKa values around 4 regarding their acid function [52,54,55,56]. These similar pKa values suggest that they are likely to form salt-like entities with maleic acid based on the ΔpKa values between these substances, which has been confirmed by the maleate systems described in the literature [35,50,51,52,53]. This establishes maleic acid as a promising candidate for reliable salt production with pharmaceutically active GABA derivatives, distinguishing it from other GRAS-list co-formers like tartaric or malic acid. These acids tend to produce unreliable phase mixtures with Baclofen and Phenibut, and malic acid forms potentially viscous residues with Pregabalin [56,57]. Other factors in crystal phase production, such as time and waste reduction, are also important. Traditionally, salts or crystalline modifications are obtained through solvent-based methods, while mechanochemistry offers a quick process with minimal solvent waste [58,59,60]. However, mechanochemically prepared systems may have higher amorphicity, more crystalline defects, or morphology changes that can affect product properties [61,62,63]. Our group observed similar findings for Baclofen and Phenibut, where multicomponent forms received through grinding or solvent-based methods showed different thermal properties in some cases [57]. Over the years, various theories on crystallization mechanisms during grinding have been proposed. These include local temperature increases inducing melt-like crystallization or local defects caused by mechanic stress, leading to particle diffusion through changes in surface energy [64,65,66,67].

In the course of the present work, salts of maleic acid (**MA**) with GABA (**1**), Gabapentin (**2**), Pregabalin (**3**), Phenibut (**4**) or Baclofen (**5**) were produced with the goal of improving API solubility. Novel phases were received for a GABA maleate (**1-MA**), a Gabapentin maleate hydrate (**2-MA•H_2_O)**, two (*rac*)-Pregabalin maleates (**(*rac*)-3-MA-I** and **(*rac*)-3-MA-II**) as well as a Phenibut maleate (**4-MA**) and known phases for Gabapentin maleate (**2-MA**), an (*S*)-Pregabalin maleate hydrate (**(*S*)-3-MA•H_2_O**) as well as a Baclofen maleate (**5-MA**) could be reproduced. We investigate the production of maleates using both grinding and solvent-based methods. The resulting substances are characterized using powder- and single-crystal X-ray diffraction (PXRD and SCXRD) as well as Fourier-transform infrared spectroscopy (FTIR). Their thermal properties are investigated via differential scanning calorimetry (DSC) and thermogravimetric analysis (TGA) in selected cases. Solubilities in an aqueous medium at 25 °C are determined through proton magnetic resonance spectroscopy (^1^H-NMR) for saturated solutions after three days. The solubilities and thermal properties of the maleates produced through solution crystallization are compared to those produced through the mechanochemical approach. The study shows that maleic acid is an excellent co-former for the simple production of API salts for the investigated compounds. Additionally, the advantages and disadvantages of the mechanochemical versus solvent-based production of these substances are discussed.

## 2. Materials and Methods

**Chemicals**: Maleic acid was purchased from TCI, GABA from J&K Scientific, Gabapentin and (*rac*)-Pregabalin from abcr, Phenibut from BLDpharm and Baclofen from Flurochem. (*S*)-Pregabalin was synthesized from (*rac*)-Pregabalin hydrate according to our previously reported approach [56].

**Maleate Preparation**: All systems **1-MA**–**5-MA** were prepared by mixing equimolar ratios of maleic acid and APIs. Solution products were grown from aqueous solution by slow evaporation of the solvent at ambient temperature. Grinding products were produced in a Retsch MM400 ball mill with 10 mL stainless steel vessels and two ZrO_2_ balls (diameter: 1 cm) via neat grinding (for **(*S*)-3-MA•H_2_O** liquid-assisted grinding with 54 μL water) at 25 Hz for 30 min. **1-MA** was produced using 5 mmol of **1** and 5 mmol amount of **MA**; **2-MA**, **2-MA•H_2_O**, **(*rac*)-3-MA-I, (*rac*)-3-MA-II** and **4-MA** using 4 mmol of APIs **2**, ***(rac)*-3** or **4** and the same amount of **MA**; **(*S*)-3-MA•H_2_O** and **5-MA** using 3mmol of ***(S)*-3** or **5** and an equivalent amount of **MA**. Attempts at producing **2-MA•H_2_O** by liquid-assisted grinding similar to **(*S*)-3-MA•H_2_O** and producing **2-MA** from solution were unsuccessful. It was not possible to successfully separate **(*rac*)-3-MA-I** and **(*rac*)-3-MA-II** under the investigated experimental conditions.

**PXRD**: Powder patterns were recorded on a Rigaku Miniflex 300 powder diffractometer with a Cu-Source and Kα radiation at 1.54184 λ in Θ/2Θ-geometry. Measurements were conducted at ambient temperature in a range of 5–50° 2Θ.

**SCXRD**: Crystals suitable for single crystal diffraction were selected under a polarized-light microscope, covered in a protective oil, and mounted on a cryo-loop. The single crystal diffraction data were recorded on a Rigaku XtaLAB Synergy S diffractometer with a Hybrid Pixel Arrow detector and a PhotonJet X-ray source using Cu-Kα radiation (λ = 1.54182 Å) at 100.0 ± 0.1 K with ω-scans. Plate-shaped colorless crystals were selected for measurement: **1-MA** (0.2 × 0.19 × 0.05 mm), **2-MA•H_2_O** (0.38 × 0.33 × 0.07 mm), **(*rac*)-3-MA-I** (0.22 × 0.11 × 0.02 mm), **(*S*)-3-MA•H_2_O** (0.52 × 0.1 × 0.06 mm), **4-MA** (0.21 × 0.17 × 0.08 mm) and **5-MA** (0.21 × 0.11 × 0.06 mm). Data reduction and absorption correction were conducted via CrysAlisPRO v. 42 software, with numerical absorption correction based on gaussian integration over a multifaceted crystal model and empirical absorption correction using spherical harmonics, implemented in SCALE3 ABSPACK scaling algorithm [68]. Structure analysis was performed by direct methods (SHELXT-2015), full-matrix least-squares refinements on F2 were done using the SHELXL2017/1 program package, and structure solution and refinements were done using Olex2-1.5 software [69,70,71]. Hydrogen atoms were freely refined except for C-H hydrogens with the following atomic displacement parameter: U_iso_(H_CH_) = 1.2 U_eq_. Figures were prepared with Mercury software v. 2022.3.0 [72]. The crystallographic data for the structures were deposited in the Cambridge Crystallographic Data Centre (CCDC-numbers 2221363–2221368) and can be obtained free of charge via www.ccdc.cam.ac.uk/data_request/cif.

**FT-IR**: Infrared spectra were recorded on a Bruker Tensor 27 Fourier transformed IR spectrometer using attenuated total reflectance mode in the range 4000 cm^−1^ to 400 cm^−1^. Samples grown from the solution were measured after evaporation of the solvent and subsequent drying at ambient temperature over two weeks for **(*S*)-3-MA•H_2_O** and **(*rac*)-3-MA** forms. Several spectra were recorded at earlier and later points in time.

**Thermal properties**: Differential scanning calorimetry was performed on a NETZSCH DSC 204F1 Phoenix device in pierced alumina crucibles at heating rates of 5 Kmin^−1^. Temperature calibration was applied, and a reference crucible with a mass of 34.728 mg was used. Crucible and sample masses were recorded, and the calculation of peak enthalpies was conducted through NETZSCH Proteus software based on measured DSC curves. Measurements of DSC samples were performed from samples that were dried at ambient conditions for two weeks after removal from the mother liquid. Thermogravimetric analysis was performed for samples **(*S*)-3-MA•H_2_O** and **(*rac*)-3-MA** on a Netzsch TG 209 F3 Tarsus with 10 Kmin^−1^ in a temperature range from 30 °C–350 °C.

**Solubilities**: Determination of substance solubilities were performed through ^1^H-NMR spectroscopy with a Bruker Avance III NMR-spectrometer at 600 MHz. Saturated solutions of crystalline material were placed in an incubator at 25 °C and moved for 60 min^−1^. Three samples of each investigated substance were left for three days under these conditions. Subsequently, 50 μL of the solution was removed from the samples and added to 450 μL of D_2_O for ^1^H-NMR measurements. Solubility was determined by the ratio of integrated solvent to substance peak. Solubility values are presented as averages over the recorded samples with the error margin according to their standard deviation.

## 3. Results

### 3.1. Structural Properties

**Scheme 1:**
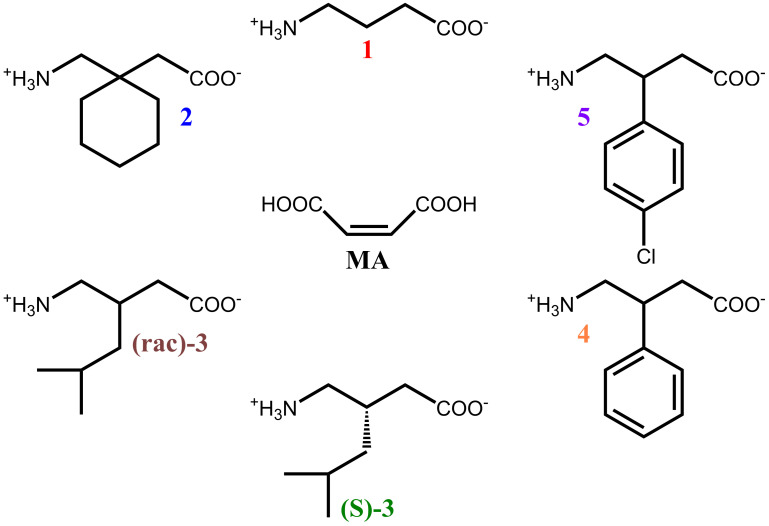
Investigated compounds maleic acid (**MA**), GABA (**1**), Gabapentin (**2**), Pregabalin (**3**), Phenibut (**4**) and Baclofen (**5**).

Maleates of GABA and its pharmaceutically active derivatives (**Scheme 1**) were produced by crystallization through solvent evaporation from an aqueous solution, and neat or liquid-assisted grinding. For most systems, single crystals were obtained through solvent evaporation, with the exception of a presumably anhydrous form of **2-MA**, which was only obtainable through neat grinding, and **(*rac*)-3-MA-II**, which occurs concomitantly with **(*rac*)-3-MA-I**, regardless of the crystallization method. Additionally, the crystallization of the enantiomerically pure (*S*)-form leads to different results than using the racemate in the case of **3**. A phase-pure maleate hydrate can be obtained by mechanochemical as well as solvent-based synthesis. However, to obtain the product through the mechanochemical route, liquid-assisted grinding must be performed as crystal synthesis of a new phase fails otherwise. **Table 1** provides an overview of the obtained products depending on the method used.

**Figure 1:**
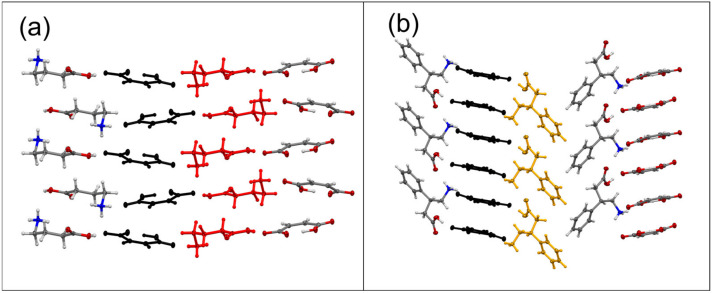
Packing in (**a**) **1-MA**, view along the a-axis and (**b**) **4-MA** view along the b-axis. Rows are highlighted according to the chosen color scheme; oxygen atoms are depicted in red, nitrogen atoms in white, carbon atoms in grey, and hydrogen atoms in white.

**Figure 2:**
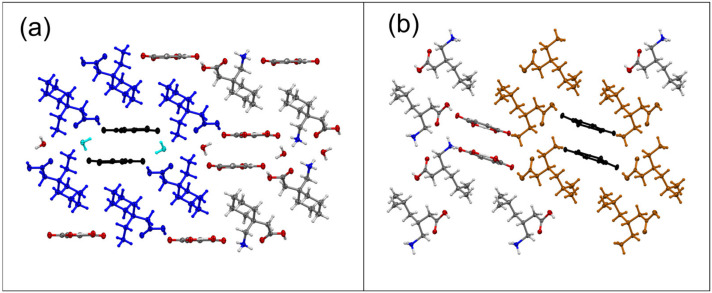
Packing in (**a**) **2-MA•H_2_O**, view along the c-axis and (**b**) **(*rac*)-3-MA** view along the b-axis. Motifs are highlighted according to the chosen color scheme; oxygen atoms are depicted in red, nitrogen atoms in white, carbon atoms in grey and hydrogen atoms in white.

**Figure 3:**
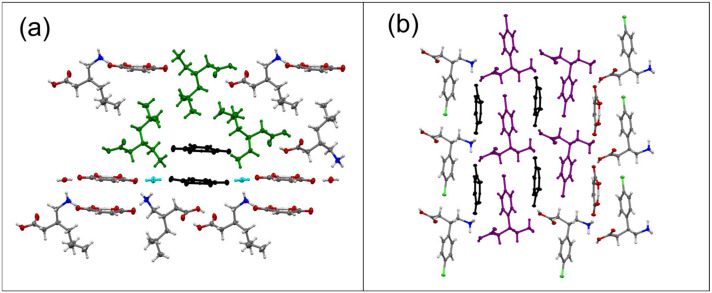
Packing in (**a**) **(*S*)-3-MA•H_2_O** view along the c-axis and (**b**) **5-MA** view along the c-axis. Motifs are highlighted according to the chosen color scheme; oxygen atoms are depicted in red, nitrogen atoms in white, carbon atoms in grey and hydrogen atoms in white.

Crystal structures of the various obtained **MA** salts differ in some respects. For example, simple rows of molecules are present in **1-MA** and **4-MA** (**Figure 1**). These follow the pattern A-B-A-B in the former, while A-B-B-A rows are formed in the latter. Hydrogen bonds (HBs) are the main attractive interaction, but in **4-MA**, edge-to-face π-interactions connect phenyl subunits. In **2-MA•H_2_O** and **(*rac*)-3-MA-I**, pairs of **MA** molecules are surrounded by API molecules, and in the case of **2-MA•H_2_O**, additional water molecules connect the **MA** pairs via HBs (**Figure 2**). The motif is more complex in **(*S*)-3-MA•H_2_O** and **5-MA** (**Figure 3**). **(*S*)-3-MA•H_2_O** breaks the encirclement of maleic acid pairs by introducing water into its lattice. Water entities and half of the **MA** molecules form a straight row, thus pushing apart the **(*S*)-3** molecules that surround **MA** pairs in **(*rac*)-3-MA-I**. In **(*S*)-3-MA•H_2_O** dimers of Pregabalinium cations are formed via carboxyl/carboxyl HBs, while in **(*rac*)-3-MA-I,** similar dimers are formed, which are connected along their GABA-subunits through carboxyl/ammonium HBs. Exclusive interaction behavior can also be observed in **5-MA**. The packing can be interpreted as either shifting rows of **5** and **MA** molecules or cavities of Baclofenium cations that are filled with **MA** entities.

**Figure 4:**
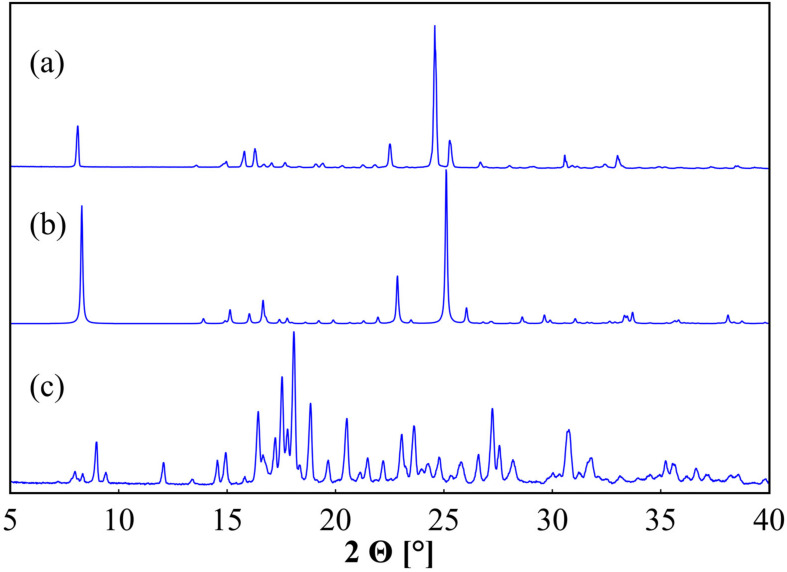
Powder patterns of **2**-based maleates. Sample of **2-MA•H_2_O** obtained by solution crystallization (**a**), simulated pattern based on single crystal data (**b**), and a sample of **2-MA** obtained by mechanochemical crystal synthesis (**c**). Powder patterns were recorded in a range of 5°–40° 2Θ.

The choice of synthesis route can have an impact on the resulting product, as well as on the physicochemical properties, the degree of crystallinity, and the quality of the sample. For **1-MA**, **(*S*)-3-MA•H_2_O**, **4-MA,** and **5-MA,** the same system is obtained regardless of the synthesis route (see Supplementary Material for **1-MA** and **5-MA**). However, different phases are obtained for **2** and **(*rac*)-3**-based maleates. Crystallization from water results in **2-MA**•**H_2_O**, while mechanochemical synthesis leads to the presumedly anhydrous **2-MA** (**Figure 4**). Attempts at liquid-assisted grinding crystallization that work for **(*S*)-3-MA•H_2_O** are unsuccessful for **2-MA**•**H_2_O** and lead to phase mixtures.

**Figure 5:**
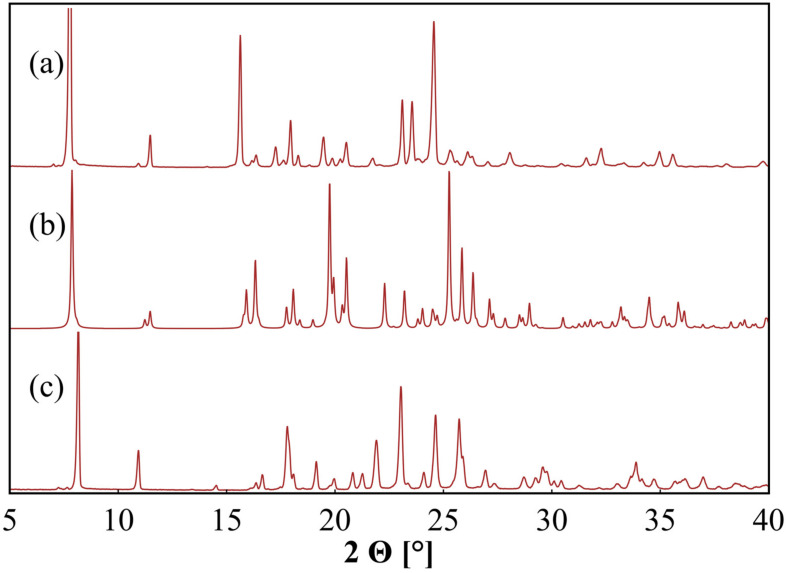
Powder patterns of **(*rac*)-3** based maleates. Sample of **(*rac*)-3-MA** obtained by solution crystallization, a larger amount of form **I** (**a**), the simulated pattern of **(*rac*)-3-MA-I** based on single crystal data (**b**), and a sample of **(*rac*)-3-MA** obtained by mechanochemical crystal synthesis, a larger amount of form **II** (**c**). Powder patterns were recorded in a range of 5°–40° 2Θ.

**Figure 6:**
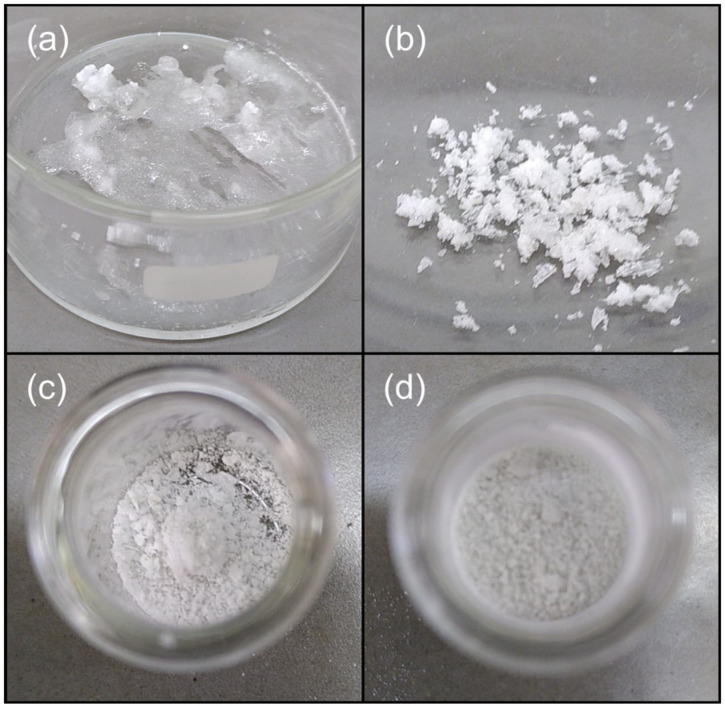
Comparison of **3** products received by solution and grinding crystallization: **(*S*)-3-MA•H_2_O** three days of drying at ambient temperature after removal from mother liquid (**a**), and produced mechanochemically (**c**), **(*rac*)-3-MA** three days of drying at ambient temperature (**b**), and **(*rac*)-3-MA** after grinding (**d**).

In the case of **(*rac*)-3-MA**, the distinction of the phases is not as straightforward as in **2-MA** systems. Crystallization via both means leads to a phase mixture, as neither diffraction pattern can be clearly assigned to simulated diffraction data from single crystal analysis (**Figure 5**). Contrary to the **2-MA** systems, the **(*rac*)-3-MA** forms undergo a phase transition over time. Interestingly, the simulated powder pattern of **(*rac*)-3-MA-I** fits better with the more commonly obtained but apparently less stable form. During the conducted experiments, it was unreliable whether a larger degree of **(*rac*)-3-MA-I** or **(*rac*)-3-MA-II** was formed in the samples. Each system was prepared thrice through solvent evaporation and mechanochemically, but only once for a mechanochemical synthesis, a larger part of **(*rac*)-3-MA-II** was formed. Given enough time, **(*rac*)-3-MA-I** converts to **(*rac*)-3-MA-II** (see Supplementary Material). Thus, it seems unlikely that **(*rac*)-3-MA-II** is a hydrate like **2-MA**•**H_2_O** or **(*S*)-3-MA•H_2_O**, and a true polymorphic conversion occurs. It is important to note that the degree of crystallinity and sample condition can greatly affect the experimental results and the properties of the material. In the case of **(*S*)-3-MA•H_2_O,** there is difficulty in obtaining a uniform, dry sample through solvent evaporation. A mixture of pasty residue and brittle crystalline material is received that keeps water residues for exceedingly long times (**Figure 6**). Even if solid **(*S*)-3-MA•H_2_O** is removed from its mother liquid and left to dry at ambient conditions, it does not become a uniformly dry substance after days or even weeks.

**Figure 7:**
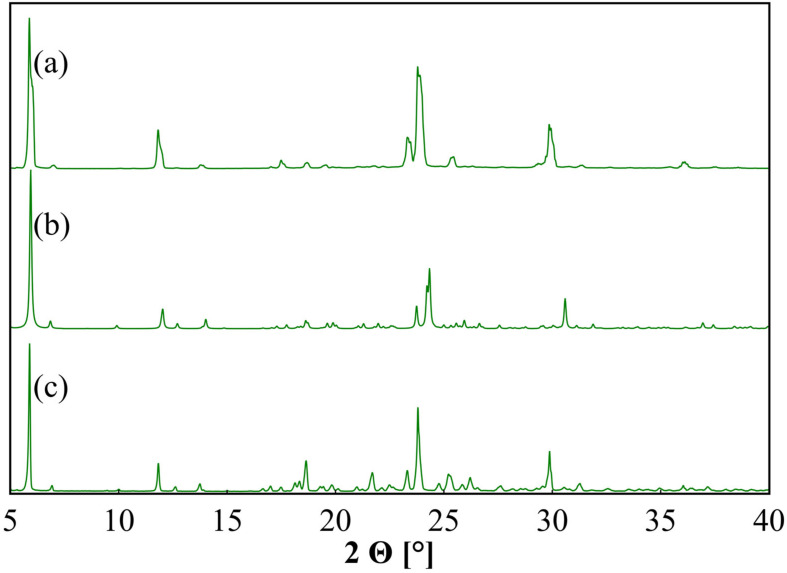
Powder patterns of **(*S*)-3** based maleates. Sample of **(*S*)-3-MA•H_2_O** obtained by solution crystallization (**a**), simulated pattern based on single crystal data (**b**), and a sample of **(*S*)-3-MA•H_2_O** obtained by mechanochemical crystal synthesis (**c**). Powder patterns were recorded in a range of 5°–40° 2Θ.

The compound can be dried under vacuum and heating, but the temperature must not be too high because melting occurs at low temperatures, as will be elaborated on in the upcoming paragraphs. Mechanochemical production with liquid-assisted grinding forms a powder of uniform quality. Additionally, powder patterns of mechanochemically produced samples show a higher degree of crystallinity by their signal resolution compared to those of samples received through solvent evaporation (**Figure 7**).

**Figure 8:**
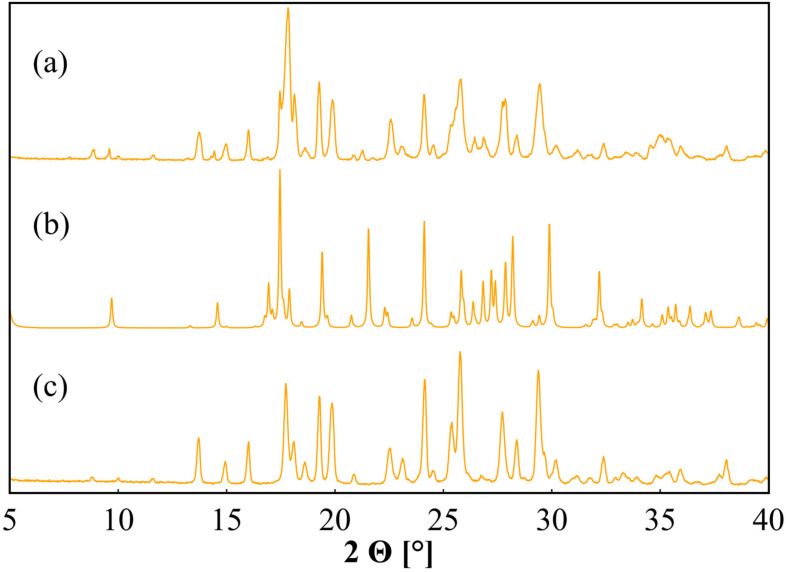
Powder patterns of **4**-based maleates. Sample of **4-MA** obtained by solution crystallization (**a**), simulated pattern based on single crystal data (**b**), and a sample of **4-MA** obtained by mechanochemical crystal synthesis (**c**). Powder patterns were recorded in a range of 5°–40° 2Θ.

In the case of **4-MA**, both synthesis routes lead to the same product. However, powder patterns received by mechanochemical and solution crystallization show bad signal resolutions and, thus, a low degree of crystallinity. Furthermore, even though the same phase is obtained through both means, not all Bragg reflections can be attributed to the simulated pattern from single crystal data (**Figure 8**). These signals cannot be assigned to precursor material **4** or **MA** either. Thus, it appears that an additional crystallization product forms here that are not yet characterized by single-crystal diffraction. Past works have shown that **4** tends to form non-pure phases more commonly, which is likely due to comparatively high lattice energy that makes the formation of multicomponent phases unfavorable [56,57].

### 3.2. Thermodynamic Properties

**Figure 9:**
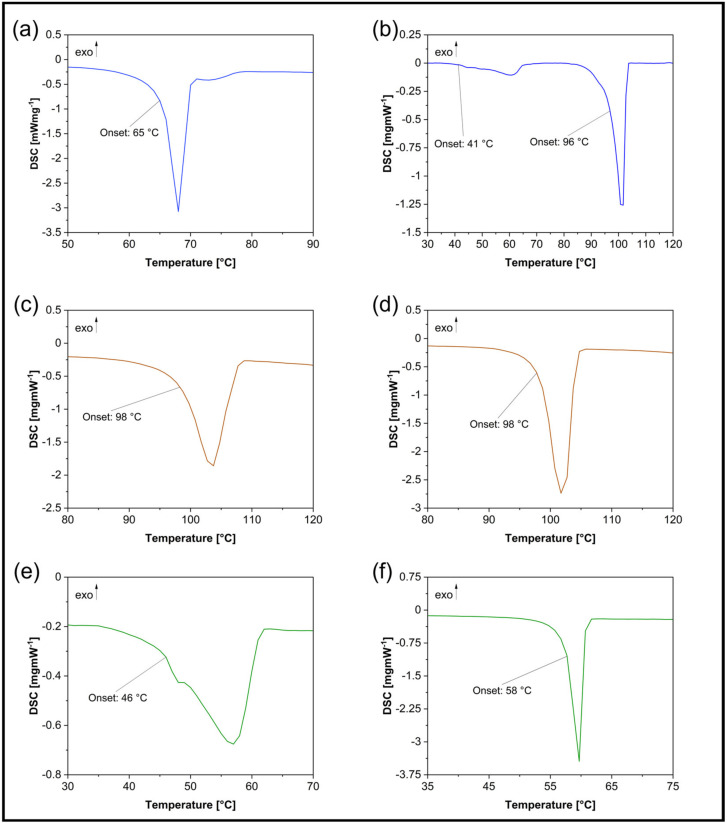
DSC-thermograms of selected **MA** systems: **2-MA•H_2_O** synthesized from solution (**a**), **2-MA** obtained mechanochemically (**b**), **(*rac*)-3-MA** synthesized from solution (**c**), and mechanochemically (**d**), **(*S*)-3-MA•H_2_O** synthesized from solution (**e**), and via grinding (**f**). For a better visibility, only the relevant temperature range is depicted.

**Figure 10:**
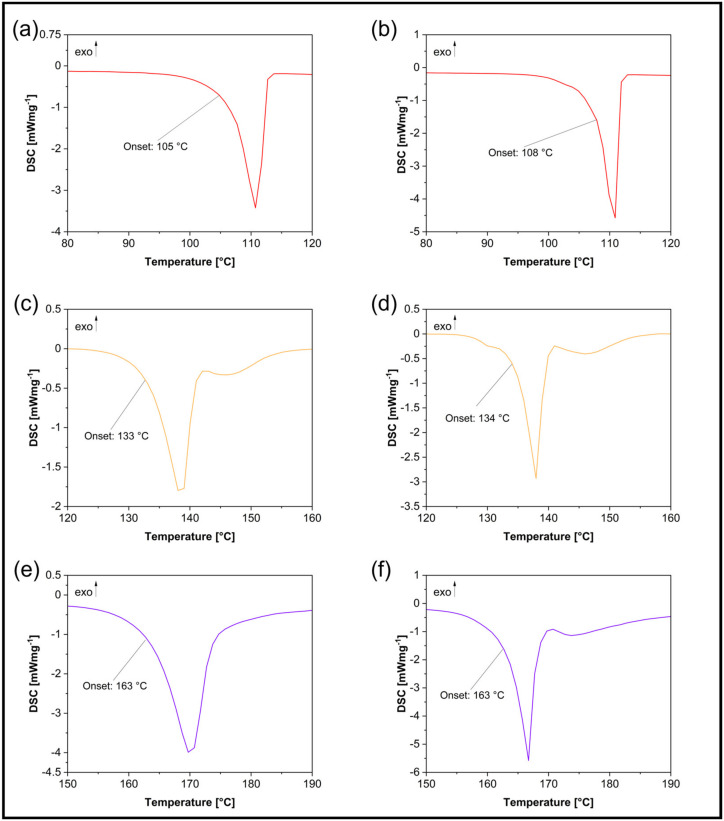
DSC-thermograms of **MA** systems showing similar thermal behavior independent of the synthetic route: **1-MA** synthesized from solution (**a**) and mechanochemically (**b**), **4-MA** synthesized from solution (**c**), and via grinding (**d**), **5-MA** synthesized from solution (**e**), and mechanochemically (**f**). For a better visibility, only the relevant temperature range is depicted.

The thermodynamic properties of the crystal phase are mostly dependent on the received species, but the crystal synthesis route and concomitant changes in sample quality play a role as well. What all systems have in common is that they do not recrystallize upon cooling in a DSC. DSC-thermograms reveal differences in melting behavior and, in some cases, indicate the presence of impurities that were not visible in PXRD and IR analyses (**Figure 9** and **Figure 10**, **Table 2**).

For instance, a DSC measurement of **2-MA** indicates the presence of poorly resolved transition signals with an onset of 41 °C, in addition to the main signal with an onset of 96 °C. Although this onset temperature is lower than that of **2-MA•H_2_O** with its well-defined melting signal and onset of 65 °C, it is still likely that hydrate impurities exist in **2-MA**. The peak positions of the small signal in **2-MA** and the melting signal in **2-MA•H_2_O** are closer to each other at onsets with 61 °C and 68 °C for the anhydrous phase and hydrate, respectively. This suggests that adsorbed water from the milling vessels was sufficient to form the hydrate, at least partially. Conversely, distinguishing between **(*rac*)-3-MA** species is more challenging. Both forms, **(*rac*)-3-MA-I** and **(*rac*)-3-MA-II**, appear to always exist concomitantly regardless of the synthesis route and have very closely related melting points. The onset for these products was determined at 98 °C, but the signal width in the solvent sample at 7 °C is nearly double that of the mechanochemical sample at 4 °C. It is possible that the large peak width in the solvent sample covers an additional signal that would otherwise be visible due to the higher content of form **I**.

This highlights how thermodynamically similar both forms are and that the difference in phase stability is minimal. The DSC signals for **(*S*)-3-MA•H_2_O** entities reveal a much larger distinction in the melting peaks, confirming the lower degree of crystallinity previously indicated in powder patterns for the solvent sample. The melting enthalpy is lowered by 34 Jg^−1^ to a value of 53 Jg^−1^ from 87 Jg^−1^ in the mechanochemically prepared product, and the onset occurs at lower temperatures with 46 °C for the solvent sample compared to 58 °C in the grinding sample. This is likely due to the higher water content still present in the sample, even after prolonged drying for two weeks, after which no evidence of excess water was present in any other sample except for **3** forms (see Supplementary Material). The early onset might indicate that dissolution in the residual water starts due to the increase in temperature.

**Figure 11:**
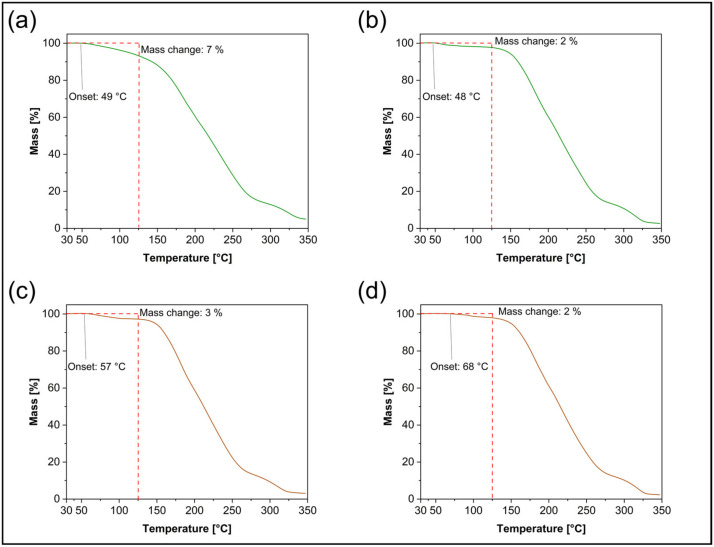
Thermogravimetric analyses of **3**-based maleates recorded after two weeks of drying at ambient conditions. A solvent sample (**a**) and a mechanochemically prepared sample (**b**) of **(*S*)-3-MA•H_2_O**, a solvent sample (**c**) and a mechanochemically prepared sample (**d**) of **(*rac*)-3-MA**. Decomposition was recorded in a range of 30 °C–350 °C. The red dotted line indicates the mass loss at 125 °C.

TG analysis was performed for **3**-based maleates (**Figure 11**). The thermal decomposition reveals a higher content of water with a mass loss of 7% at 125 °C in **(*S*)-3-MA•H_2_O** compared to the other **3** maleates that show losses of only 2–3%. The compound can be dried forcefully at 40 °C in a vacuum atmosphere, but the drying procedure takes a long time; as such, a low drying temperature must be chosen due to the low melting point (see Supplementary Material). The TG analysis further confirms that **(*rac*)-3-MA** forms **I** and **II** behave similarly regarding their thermal properties. While the solvent sample starts to lose water at a lower temperature of 57 °C compared to 68 °C in the mechanochemical sample, the mass loss is only at 3% and 2%, respectively. The crystal structure of **(*rac*)-3-MA-I** does not incorporate lattice water, so the slightly higher water content in the solvent sample likely stems from excess water from the solution. In the grinding sample, this water might be explained by lattice water of **(*rac*)-3•H_2_O**, with which it was prepared.

Thermal analyses of the less distinctive maleates reveal some significant characteristics as well. **1-MA** shows slightly different melting points for the distinctive synthesis routes, with onsets of 105 °C and 108 °C for solution crystallization and grinding, respectively. However, the peak positions are identical at 111 °C, and as there are no variations in powder patterns or IR-spectra, the most probable explanation is a more uniform dispersion of crystallite sizes in the mechanochemical sample, leading to a sharper melting signal. For **4-MA**, the presence of impurities is confirmed. Next to intense melting signals with onsets of 133 °C and 134 °C, there are smaller signals in a very close range to the main one. Both samples show a small phase transition that partly overlaps with the large melting peak, starting at ca. 142 °C. In the mechanochemically prepared sample, an even smaller phase transition occurs at about 127 °C. As the melting signal in the solvent sample is again dispersed over a larger temperature area, it seems possible that it covers the smaller signal here. However, even though no pure phase is obtainable for **4-MA** by either synthesis route, both means lead to the same, impure end. More surprising is the result for **5-MA**. Diffraction patterns and IR spectra show no distinctive features regardless of the crystallization method, except for a worse Bragg reflection resolution for the grinding sample. Still, an additional phase transition after the main melting signal with an onset of 163 °C in both samples is visible in the grinding product at ca. 170 °C. It seems possible this signal is again covered in the solvent sample, as the signal peak area at 8 °C is double that of the milling one at 4 °C.

**Figure 12:**
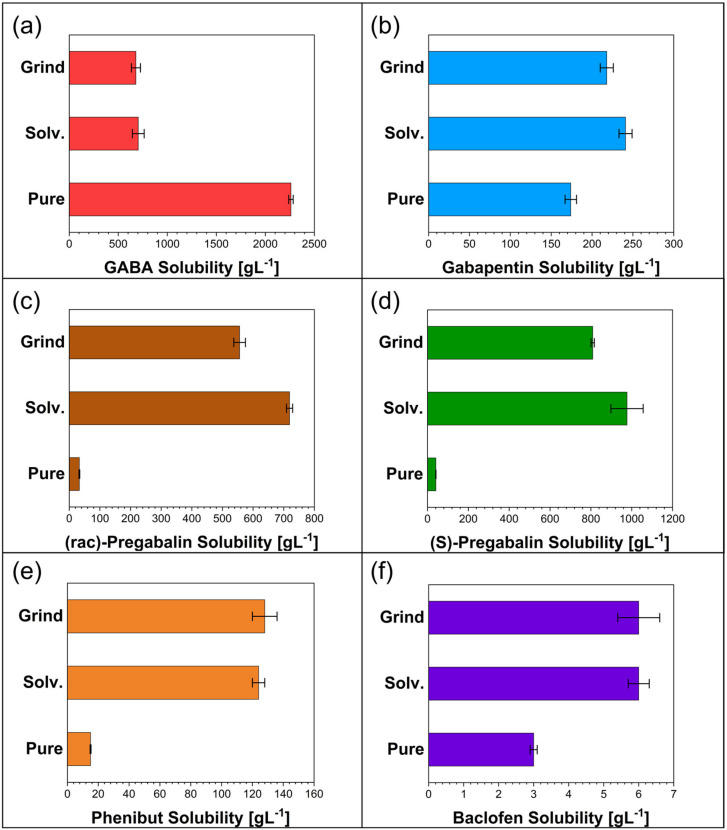
Recorded solubilities of the investigated samples in gL-1 regarding their API solubility of (**a**) **1**, (**b**) **2**, (**c**) **(*rac*)-3**, (**d**) **(*S*)-3**, (**e**) **4**, and (**f**) **5**-based compounds. Solubilities were determined by preparation of saturated dispersions of target compounds in an aqueous solution at 25 °C after three days through ^1^H-NMR spectroscopy against the water signal.

Solubility in an aqueous medium for all substances was determined through ^1^H-NMR spectroscopy. In the past, various studies have shown that 1H-NMR can be used as an effective tool in quantitatively assessing sample content in a solvent medium [73,74,75,76]. While high-performance liquid chromatography can offer even more accurate results, ^1^H-NMR-based evaluation of solubility is simple and quick and can provide great accuracy as well. The determined solubilities for the different maleates show that increases are always observed, except for **1-MA** (**Figure 12**, **Table 3**). However, **1** alone is exceedingly soluble in water with a determined solubility of 2261 ± 23 gL^−1^. On the other hand, the solubility of **MA** was measured as 687 ± 44 gL^−1^. Solubilities of multicomponent crystalline species mostly fall in between that of their co-formers, so it appears this is the likely explanation.

The largest discrepancies occur between different phases for **2** and **(*rac*)-3**-based systems and for **(*S*)-3** milling and solution crystallization forms. The solubilities of **2-MA•H_2_O** and **2-MA** only differ slightly, and they are just outside each other’s error margin with 241 ± 8 gL^−1^ and 218 ± 8 gL^−1^, respectively. The difference is even higher between **(*rac*)-3-MA** obtained from solution and by grinding with 719 ± 10 gL^−1^ for a sample that contains a larger degree of form **I** and 556 ± 19 gL^−1^ for a sample that contains more form **II**. These values show that both forms increase the content of **(*rac*)-3** in solution substantially, but they are influenced by each other’s presence and, thus, it is difficult to say whether form **I** could be even more soluble and form **II** may be slightly less so. The largest influence of the crystal synthesis route on a system that exhibits the same diffraction pattern is present for **(*S*)-3-MA•H_2_O**. The solubility of the solvent product with 977 ± 79 gL^−1^ is decisively higher than that of the grinding product with 809 ± 8 gL^−1^. As was shown, this can be explained by the sample condition. The solvent product shows a higher degree of amorphicity and contains a higher amount of water from the start. This might ease the hydration of **(*S*)-3** molecules and thereby increase the dissolution speed compared to mechanochemically produced **(*S*)-3-MA•H_2_O**. All solubilities were determined from three samples stored for three days at 25 °C under slight shaking. However, subsequent observation of **(*S*)-3-MA•H_2_O** shows that samples dissolve further after a longer period of time. The addition of more solid material led to an even higher content of dissolved **(*S*)-3-MA•H_2_O**. The result is a strongly viscous goo. This observation highlights how viscid residue formation makes it so difficult to obtain a uniform product of **(*S*)-3-MA•H_2_O** from the solution. For **4-MA** and **5-MA**, uniform increases in solubility were observed, with no significant differences between products obtained via mechanochemical and solvent evaporation crystallization. However, the solubility of **5-MA** remains relatively low at 6 gL^−1^.

## 4. Discussion

The results of this work can be summarized into two key aspects. The first one is that maleic acid can serve as an excellent co-former for GABA-related APIs and shows great potential to enhance their solubility. In the case of the presented systems, the solubility of Baclofen and Gabapentin could be increased by twofold, of Phenibut by eightfold, and (*S*)-Pregabalin, as well as (*rac*)-Pregabalin by 21- and 23-fold, respectively. However, in many cases, there are multiple phases obtainable, sometimes dependent and sometimes independent of the synthesis route. The examples of **2**, **(*rac*)-3,** and **4**-based systems show that phase control can be challenging. **2-MA•H_2_O** and **2-MA** can be produced with satisfactory reliability from an aqueous solution or through grinding, respectively. Conversely, **(*rac*)-3-MA** species are obtained as a phase mixture, regardless of the crystallization route under the investigated conditions. This is evidenced by their distinctive powder patterns, as well as large solubility discrepancies. However, it is still possible to clearly assign the **(*rac*)-3-MA-I** forms Bragg reflections to the simulated diffraction pattern from single crystal data, which in conjunction with the distinctiveness of the grinding pattern and solubility differences, is why the authors feel confident in claiming another polymorph **(*rac*)-3-MA-II**. The same cannot be said about **4-MA**, where the powder patterns of the solubility and grinding product fit acceptably among each other. Discrepancies could be ascribed to the synthesis method and corresponding differences in crystallinity, but neither pattern fits very well with the one simulated from single crystal data. This could imply an additional phase, but neither thermograms nor solubilities show clear evidence for distinctive behavior, such as in **(*rac*)-3-MA-I** and **-II**. Even though produced and simulated patterns do not fit, because of the similarities in patterns of solubility and milling product and lacking distinctiveness in physicochemical properties, it remains uncertain whether an additional polymorph is present for **4-MA**. As both recorded powder patterns of solubility and grinding product stem from their respective bulk phases, it could be possible that the recorded single crystal structure is a polymorphic impurity. However, too little material for a powder measurement of this system was produced, and it appears to be impossible to gain a good sample of the potential other entity under the investigated conditions.

The second and, an arguably more valuable point that can be made is that it is worthwhile to investigate different synthesis routes for various API maleates. For **1**, **4** and **5** maleates, the synthesis method does not matter, and a similar result is obtainable either way. Regarding **2-** and **(*rac*)-3**-based systems, different phases are obtained depending on which crystallization method is used; results for the former are reliable, while for the latter phase, mixtures are received. The highest impact of the synthesis route is present in the case of **(*S*)-3-MA•H_2_O**. Contrary to its **(*rac*)-3** based counterpart, the same phase is always observable by diffraction and IR analyses of grinding and solvent-evaporation products. Still, large discrepancies were recorded concerning their thermodynamic properties. The mechanochemical product is a powder that melts higher and with a sharper melting signal, but the maximum solubility of the product is lower. The solvent product is of a moist and pasty consistency, melts lower and irregularly, but shows higher overall solubility after the same time frame compared to the grinding sample. Even if maximum solubility is higher in the solution form, there is a clear advantage of the milling route in this case. The processability of the milling product is much better, and why should time and energy be spent on drying pasty solvent grown **(*S*)-3-MA•H_2_O** when it could be produced mechanochemically instantly and in uniform condition? Both solubilities are very high compared to pure **(*S*)-3,** regardless.

It was shown that maleic acid is an interesting candidate for the soluble salt formation of GABA-derived APIs. The formation of a new phase is facile and occurs readily. Phase purity can be problematic, but it is not necessarily, depending on the API. As especially Gabapentin and Pregabalin continue to be commercial blockbusters, this study aims to highlight the usefulness of maleate formation for potential successors in the future.

It was furthermore demonstrated that the crystal synthesis method can greatly affect the received maleate phase and quality. A comparison of solvent and mechanochemical crystallization regarding chosen categories is presented in **Table 4**.

Regarding time consumption, mechanochemical crystal synthesis is clearly better than solvent crystallization. Grinding crystallizations for the investigated systems take place in the minute range, while crystallization from an aqueous solution can take days to occur. Attempting to forcefully induce crystallization from solution by heating evaporation can lead to a bad product, for example, in very well-soluble Pregabalin species, and still costs more time and energy. However, in terms of the other categories, the comparison is more of a stalemate. The phase reliability of the received maleates was good or bad in the respective cases regardless of the crystallization procedure. Here, it is more important which phase should actually be produced, and thus it is situational which method leads to the better results. The last compared item is product quality. The authors assign a slightly better result to mechanochemical crystallization here foremost because of the **(*S*)-3** example. Furthermore, maleates that are produced mechanochemically are, by default, more uniform powders, equally dry and with similar crystallite sizes depending on the grinding process. However, the crystallinity is often lower compared to solvent-based crystallization, and single crystals cannot be obtained. Conclusively, it should be evaluated which method can lead to the desired product in a reliable manner. Mechanochemical crystallization should always be considered, as it can potentially be a very fast method to receive a product of uniform quality.

## Figures and Tables

**Table 1:** Overview on the conducted crystallization methods for **1**–**5** maleates.

Sample	Solvent Cryst.	Neat Grind.	Liquid-Assisted Grind.
**1-MA**	pure phase	pure phase	not conducted
**2-MA**	pure 2-MA•H_2_O	pure phase	mixture with 2-MA•H_2_O
**2-MA•H_2_O**	pure phase	pure 2-MA	mixture with 2-MA
**(*rac*)-3-MA-I**	more (*rac*)-3-MA-I	more (*rac*)-3-MA-II	not conducted
**(*rac*)-3-MA-II**	more (*rac*)-3-MA-I	more (*rac*)-3-MA-II	not conducted
**(*S*)-3-MA•H_2_O**	pure phase	no new phase	pure phase
**4-MA**	impure phase	impure phase	not conducted
**5-MA**	pure phase	pure phase	not conducted

**Table 2:** Overview of the recorded DSC parameters for all investigated samples. Temperature values have been rounded to full numbers.

Sample	Melting Enthalpy [Jg^−1^]	Onset [°C]	End [°C]	Peak [°C]	Peak Width [°C]	Peak Height [mWmg^−1^]
**1-MA**	176	105	113	111	5	3.242
**1-MA_M**	178	108	112	111	3	4.673
**2-MA•H_2_O**	135	65	70	68	3	2.864
**2-MA_M (Peak 1)**	14	41	65	61	16	0.095
**2-MA_M (Peak 2)**	85	96	103	101	5	1.345
**(*rac*)-3-MA**	125	98	107	104	7	1.619
**(*rac*)-3-MA_M**	132	98	104	102	4	2.610
**(*S*)-3-MA•H_2_O**	53	46	61	57	13	0.454
**(*S*)-3-MA•H_2_O_M**	87	58	61	60	2	3.439
**4-MA**	165	133	141	139	6	1.850
**4-MA_M**	181	134	139	138	4	2.945
**5-MA**	376	163	174	170	8	3.708
**5-MA_M**	362	163	168	167	4	5.252

**Table 3:** Solubility values, error margins and decreases or increases of API solubilities in the investigated maleates.

Sample	Solubility [gL^−1^]	Error [%]	Error [gL^−1^]
**MA**	687	6	44
**1**	2261	1	23
**2**	174	4	7
**(*rac*)-3•H_2_O**	33	4	1.3
**(*S*)-3**	41	2	0.7
**4**	15	2	0.3
**5**	3	3	0.1
**1-MA**	704	8	60
**1-MA_M**	680	7	46
**2-MA•H_2_O**	241	3	8
**2-MA_M**	218	4	8
**(*rac*)-3-MA**	719	1.4	10
**(*rac*)-3-MA_M**	556	3	19
**(*S*)-3-MA•H_2_O**	977	8	79
**(*S*)-3-MA•H_2_O_M**	809	1	8
**4-MA**	124	3	4
**4-MA_M**	128	6	8
**5-MA**	6	4	0.3
**5-MA_M**	6	10	0.6

**Table 4:** Comparison of the solvent and mechanochemical crystallization regarding quickness, reliability of the received phase, and quality of the product in terms of further processing capabilities. A “+” symbol suggests a better result, a “-“ symbol a worse result and a “=” symbol an equal result.

Category	Solvent Cryst.	Mechanochemical Cryst.
**Quickness**	-	+
**Phase reliability**	=	=
**Product quality**	= (slight +)	= (slight -)

## Data Availability

Not applicable.

## Supplementary Materials

The following supporting information can be downloaded at: https://www.mdpi.com/article/10.3390/ma16062242/s1, Figure S1: Powder pattern comparison of (a) recorded GABA pattern, (b) GABA maleate as produced from aqueous solution, (c) GABA maleate pattern simulated by single crystal data, (d) GABA maleate as produced by neat grinding, and (e) recorded maleic acid pattern. A 2Θ range from 5–40° is depicted. Figure S2: FT-IR spectra of (a) GABA, (b) GABA maleate as produced from aqueous solution, (c) GABA maleate as produced by neat grinding and (d) maleic acid. Spectra are recorded between 4000 cm^−1^–400 cm^−1^. Figure S3: Powder pattern comparison of (a) recorded Gabapentin pattern, (b) Gabapentin maleate hydrate as produced from aqueous solution, (c) Gabapentin maleate hydrate pattern simulated by single crystal data with hkl = 0 4 0 and March–Dollase parameter of 0.5, (d) Gabapentin maleate as produced by neat-grinding, and (e) recorded Gabapentin pattern. A 2Θ range from 5–40° is depicted. Figure S4: Powder pattern comparison of (a) recorded Gabapentin pattern, (b) Gabapentin maleate as produced by neat grinding, (c) Gabapentin maleate hydrate and maleate mixture as produced by liquid-assisted grinding, (d) Gabapentin maleate hydrate as produced from aqueous solution and (e) recorded maleic acid pattern. A 2Θ range from 5–40° is depicted. Figure S5: FT-IR spectra of (a) Gabapentin, (b) Gabapentin maleate hydrate as produced from aqueous solution, (c) Gabapentin maleate as produced by neat grinding and (d) maleic acid. Spectra are recorded between 4000 cm^−1^–400 cm^−1^. Figure S6: Powder pattern comparison of (a) recorded (rac)-Pregabalin pattern, (b) (rac)-Pregabalin maleate as produced from aqueous solution, (c) (rac)-Pregabalin maleate I pattern simulated by single crystal data with hkl = 4 15 2 and March–Dollase parameter of 4, (d) (rac)-Pregabalin maleate pattern produced via neat grinding with higher content of II, and (e) recorded maleic acid pattern. A 2Θ range from 5–40° is depicted. Figure S7: Various recorded powder patterns of solvent grown (a), (b) as well as mechanochemically prepared (d), (e) (rac)-Pregabalin maleates compared to the once received phase containing more of form II (c). Figure S8: FT-IR spectra of (a) (rac)-Pregabalin hydrate, (b) (rac)-Pregabalin maleate as produced from aqueous solution, (c) (rac)-Pregabalin maleate as produced by neat grinding containing more of form II and (d) maleic acid. Spectra are recorded between 4000 cm^−1^–400 cm^−1^. Figure S9: FT-IR spectra of (a) (rac)-Pregabalin maleate as produced from aqueous solution after three days drying, (b) (rac)-Pregabalin maleate as produced from aqueous solution, two weeks drying, (c) (rac)-Pregabalin maleate as produced from aqueous solution, two weeks drying and subsequent vacuum drying at 40 °C for 2 h, and (d) (rac)-Pregabalin maleate as produced by neat grinding containing more of form II. Spectra are recorded between 4000 cm^−1^–400 cm^−1^. Figure S10: Powder pattern comparison of (a) recorded (S)-Pregabalin pattern, (b) (S)-Pregabalin maleate hydrate as produced from aqueous solution, (c) (S)-Pregabalin maleate hydrate pattern simulated by single crystal data with hkl = 0 1 1 and March–Dollase parameter of 2, (d) (S)-Pregabalin maleate hydrate as produced by liquid-assisted grinding and (e) recorded maleic acid pattern. A 2Θ range from 5–40° is depicted. Figure S11: Powder pattern comparison of (a) recorded (S)-Pregabalin pattern, (b) (S)-Pregabalin maleate hydrate as produced by liquid-assisted grinding, (c) (S)-Pregabalin maleate pattern produced by neat grinding and (d) recorded maleic acid pattern. A 2Θ range from 5–40° is depicted. Figure S12: FT-IR spectra of (a) (S)-Pregabalin, (b) (S)-Pregabalin maleate hydrate as produced from aqueous solution, (c) (S)-Pregabalin maleate hydrate as produced by liquid-assisted grinding and (d) maleic acid. Spectra are recorded between 4000 cm^−1^–400 cm^−1^. Figure S13: FT-IR spectra of (a) (S)-Pregabalin maleate hydrate as produced from aqueous solution, three days drying, (b) (S)-Pregabalin maleate as produced from aqueous solution, vacuum drying and (c) (S)-Pregabalin maleate hydrate as produced by liquid-assisted grinding. Spectra are recorded between 4000 cm^−1^–400 cm^−1^. Figure S14: Powder pattern comparison of (a) recorded Phenibut pattern, (b) Phenibut maleate as produced from aqueous solution, (c) Phenibut maleate pattern simulated by single crystal data with hkl = 1 8 1 and March–Dollase parameter of 0.65, (d) Phenibut maleate as produced by neat grinding and (e) recorded maleic acid pattern. A 2Θ range from 5–40° is depicted. Figure S15: FT-IR spectra of (a) Phenibut, (b) Phenibut maleate as produced from aqueous solution, (c) Phenibut maleate as produced by neat grinding and (d) maleic acid. Spectra are recorded between 4000 cm^−1^–400 cm^−1^. Figure S16: Powder pattern comparison of (a) recorded Baclofen pattern, (b) Baclofen maleate as produced from aqueous solution, (c) Baclofen maleate pattern simulated by single crystal data with hkl = 0 0 7 and March–Dollase parameter of 4, (d) Baclofen maleate as produced by neat grinding and (e) recorded maleic acid pattern. A 2Θ range from 5–40° is depicted. Figure S17: FT-IR spectra of (a) Baclofen, (b) Baclofen maleate as produced from aqueous solution, (c) Baclofen maleate as produced by neat grinding and (d) maleic acid. Spectra are recorded between 4000 cm^−1^–400 cm^−1^. Figure S18: Depiction of the asymmetric unit in each compound that could be characterized by SCXRD: (a) GABA maleate, (b) Gabapentin maleate hydrate, (c) (rac)-Pregabalin maleate, (d) (S)-Pregabalin maleate hydrate, (e) Phenibut maleate, and (f) Baclofen maleate. Carbon atoms are depicted in grey, hydrogen atoms in white, nitrogen atoms in blue, oxygen atoms in red, and chlorine atoms in green. Hydrogen bonds occurring in the asymmetric units are shown as light blue dotted lines. Figure S19: ^1^H-NMR spectrum of GABA recorded in D_2_O at 600 MHz. Figure S20: ^1^H-NMR spectrum of Gabapentin recorded in D_2_O at 600 MHz. Figure S21: ^1^H-NMR spectrum of (S)-Pregabalin recorded in D_2_O at 600 MHz. Also represents (rac)-Pregabalin. Figure S22: ^1^H-NMR spectrum of Phenibut recorded in D_2_O at 600 MHz. Figure S23: ^1^H-NMR spectrum of Baclofen recorded in D_2_O at 600 MHz. Figure S24: ^1^H-NMR spectrum of GABA maleate recorded in D_2_O at 600 MHz. Sample grown from solution. Figure S25: ^1^H-NMR spectrum of Gabapentin maleate hydrate recorded in D_2_O at 600 MHz. Figure S26: ^1^H-NMR spectrum of Gabapentin maleate recorded in D_2_O at 600 MHz. Figure S27: ^1^H-NMR spectrum of (rac)-Pregabalin maleate recorded in D_2_O at 600 MHz. Sample grown from solution. Figure S28: ^1^H-NMR spectrum of (rac)-Pregabalin maleate recorded in D_2_O at 600 MHz. Sample grown via grinding. Figure S29: ^1^H-NMR spectrum of (S)-Pregabalin maleate hydrate recorded in D_2_O at 600 MHz. Sample grown from solution. Figure S30: ^1^H-NMR spectrum of (S)-Pregabalin maleate hydrate recorded in D_2_O at 600 MHz. Sample grown via grinding. Figure S31: ^1^H-NMR spectrum of Phenibut maleate recorded in D_2_O at 600 MHz. Sample grown from solution. Figure S32: ^1^H-NMR spectrum of Baclofen maleate recorded in D_2_O at 600 MHz. Sample grown from solution. Table S1: Single crystal measurement details for GABA maleate. Table S2: Single crystal measurement details for Gabapentin maleate hydrate. Table S3: Single crystal measurement details for (rac)-Pregabalin maleate. Table S4: Single crystal measurement details for (S)-Pregabalin maleate hydrate. Table S5: Single crystal measurement details for Phenibut maleate. Table S6: Single crystal measurement details for Baclofen maleate. Table S7: Solubilities of GABA and its derivatives on their own and in the form of the investigated maleates and their standard deviations. The integral borders for the product peaks used for solubility calculations of samples S1–S3 are given.

## References

[1] Jornada D.H., dos Santos Fernandes G.F., Chiba D.E., de Melo T.R.F., dos Santos J.L., Chung M.C. (2015). The Prodrug Approach: A Successful Tool for Improving Drug Solubility. Molecules.

[2] Antraygues K., Maingot M., Schellhorn B., Trebosc V., Gitzinger M., Deprez B., Defert O., Dale G.E., Bourotte M., Lociuro S. (2022). Design and synthesis of water-soluble prodrugs of rifabutin for intraveneous administration. Eur. J. Med. Chem..

[3] Liu J., Wang W., Wang C., Zhang L., Zhang X., Liu S., Xu Y., Wang H., Dai Q., Liu C. (2022). Discovery of Antibacterial Contezolid Acefosamil: Innovative O-Acyl Phosphoramidate Prodrug for IV and Oral Therapies. ACS Med. Chem. Lett..

[4] Sanches B.M.A., Ferreira E.I. (2019). Is prodrug design an approach to increase water solubility?. Int. J. Pharm..

[5] Yu Y., Sun L., Tang Y., Zhu H., Wang H., Xiao H., Wang F., Tao W. (2022). Preparation of cisplatin delivery calcium phosphate nanoparticles using poly(Pt(IV) prodrug) as the payload. Mater. Today Commun..

[6] Patel V.R., Agrawal Y.K. (2011). Nanosuspension: An approach to enhance solubility of drugs. J. Adv. Pharm. Technol. Res..

[7] Pandey N., Bohra B.S., Tiwari H., Pal M., Negi P.B., Dandapat A., Mehta S., Sahoo N.G. (2022). Development of biodegradable chitosan/graphene oxide nanocomposite via spray drying method for drug loading and delivery application. J. Drug Deliv. Sci. Technol..

[8] Nora G.-I., Venkatasubramanian R., Strindberg S., Siqueira-Jørgensen S.D., Pagano L., Romanski F.S., Swarnakar N.K., Rades T., Müllertz A. (2022). Combining lipid based drug delivery and amorphous solid dispersions for improved oral drug absorption of a poorly water-soluble drug. J. Control. Release.

[9] Khan A.A., Akhtar S., Yadav Y., Akhtar A., Alelwani W., Bannunah A.M., Mahmood S. (2022). Lopinavir-Loaded Self-Nanoemulsifying Drug Delivery System for Enhanced Solubility: Development, Characterisation and Caco-2 Cell Uptake. Curr. Drug Deliv..

[10] Pignatello R., Corsaro R., Bonaccorso A., Zingale E., Carbone C., Musumeci T. (2022). Soluplus^®^ polymeric nanomicelles improve solubility of BCS-class II drugs. Drug Deliv. Transl. Res..

[11] Petitprez J., Legrand F.-X., Tams C., Pipkin J.D., Antle V., Kfoury M., Fourmentin S. (2022). Huge solubility increase of poorly water-soluble pharmaceuticals by sulfobutylether-β-cyclodextrin complexation in a low-melting mixture. Environ. Chem. Lett..

[12] Loftsson T. (2017). Drug solubilization by complexation. Int. J. Pharm..

[13] Kaur J., Singla P., Kaur I. (2022). Labrasol mediated enhanced solubilization of natural hydrophobic drugs in Pluronic micelles: Physicochemical and in vitro release studies. J. Mol. Liq..

[14] Dadej A., Woźniak-Braszak A., Bilski P., Piotrowska-Kempisty H., Józkowiak M., Pawełczyk A., Dadej D., Łażewska D., Jelińska A. (2022). Improved solubility of lornoxicam by inclusion into SBA-15: Comparison of loading methods. Eur. J. Pharm. Sci..

[15] Hancock B.C., Parks M. (2000). What is the true solubility advantage for amorphous pharmaceuticals?. Pharm. Res..

[16] Serajuddin A.T.M. (2007). Salt formation to improve drug solubility. Adv. Drug Deliv. Rev..

[17] Good D.J., Rodríguez-Hornedo N. (2009). Solubility Advantage of Pharmaceutical Cocrystals. Cryst. Growth Des..

[18] Elder D.P., Holm R., de Diego H.L. (2013). Use of pharmaceutical salts and cocrystals to address the issue of poor solubility. Int. J. Pharm..

[19] Jindal A., Prashar M., Dureja J., Dhingra N., Chadha K., Karan M., Chadha R. (2022). Pharmaceutical Cocrystals of Famotidine: Structural and Biopharmaceutical Evaluation. J. Pharm. Sci..

[20] Lenschow I.C.S., Bazzo G.C., Zétola M., Stulzer H.K., Soares L., Pezzini B.R. (2022). Ball-milled valsartan and its combination with mannitol: The case of drug polyamorphism. J. Therm. Anal. Calorim..

[21] Vemuri V.D., Lankalapalli S., Chandra Reddy Guntaka P. (2022). Posaconazole-amino acid cocrystals for improving solubility and oral bioavailability while maintaining antifungal activity and low In vivo toxicity. J. Drug Deliv. Sci. Technol..

[22] Wang Z., Chen X., Li D., Bai E., Zhang H., Duan Y., Huang Y. (2022). Platensimycin-berberine chloride co-amorphous drug system: Sustained release and prolonged half-life. Eur. J. Pharm. Biopharm..

[23] Nechipadappu S.K., Swain D. (2023). Combined synthetic and solubility aspects of orotate salt of bilastine. J. Mol. Struct..

[24] Liu L., An Q., Zhang Y., Sun W., Li J., Feng Y., Geng Y., Cheng G. (2022). Improving the solubility, hygroscopicity and permeability of enrofloxacin by forming 1:2 pharmaceutical salt cocrystal with neutral and anionic co-existing p-nitrobenzoic acid. J. Drug Deliv. Sci. Technol..

[25] Neelam U.K., Daveedu B., Ambabhai V.N., Siripragada M.R., Kumar S.R., Balasubramanian S. (2023). Physicochemical aspects and comparative analysis of Voxelotor and its salt and cocrystal. J. Mol. Struct..

[26] Bethune S.J., Huang N., Jayasankar A., Rodríguez-Hornedo N. (2009). Understanding and Predicting the Effect of Cocrystal Components and pH on Cocrystal Solubility. Cryst. Growth Des..

[27] Banik M., Gopi S.P., Ganguly S., Desiraju G.R. (2016). Cocrystal and Salt Forms of Furosemide: Solubility and Diffusion Variations. Cryst. Growth Des..

[28] Kruger C. (2016). The relevance of international assessments to GRAS determinations. Regul. Toxicol. Pharmacol..

[29] Martins F.T., Paparidis N., Doriguetto A.C., Ellena J. (2009). Crystal Engineering of an Anti-HIV Drug Based on the Recognition of Assembling Molecular Frameworks. Cryst. Growth Des..

[30] Mittapalli S., Mannava M.K.C., Khandavilli U.B.R., Allu S., Nangia A. (2015). Soluble Salts and Cocrystals of Clotrimazole. Cryst. Growth Des..

[31] Zhao Y., Sun B., Jia L., Wang Y., Wang M., Yang H., Qiao Y., Gong J., Tang W. (2020). Tuning Physicochemical Properties of Antipsychotic Drug Aripiprazole with Multicomponent Crystal Strategy Based on Structure and Property Relationship. Cryst. Growth Des..

[32] Voronin A.P., Surov A.O., Churakov A.V., Parashchuk O.D., Rykounov A.A., Vener M.V. (2020). Combined X-ray Crystallographic, IR/Raman Spectroscopic, and Periodic DFT Investigations of New Multicomponent Crystalline Forms of Anthelmintic Drugs: A Case Study of Carbendazim Maleate. Molecules.

[33] Djaló M., Cunha A.E.S., Luís J.P., Quaresma S., Fernandes A., André V., Duarte M.T. (2021). Sparfloxacin Multicomponent Crystals: Targeting the Solubility of Problematic Antibiotics. Cryst. Growth Des..

[34] Sun N., Avdeef A. (2011). Biorelevant pK(a) (37 °C) predicted from the 2D structure of the molecule and its pK(a) at 25 °C. J. Pharm. Biomed. Anal..

[35] Childs S.L., Stahly G.P., Park A. (2007). The salt-cocrystal continuum: The influence of crystal structure on ionization state. Mol. Pharm..

[36] Bowery N.G., Smart T.G. (2006). GABA and glycine as neurotransmitters: A brief history. Br. J. Pharmacol..

[37] Bown A.W., Shelp B.J. (2016). Plant GABA: Not Just a Metabolite. Trends Plant Sci..

[38] Enna S.J., McCarson K.E. (2006). The role of GABA in the mediation and perception of pain. Adv. Pharmacol..

[39] Gottesmann C. (2002). GABA mechanisms and sleep. Neuroscience.

[40] Kalueff A.V., Nutt D.J. (2007). Role of GABA in anxiety and depression. Depress. Anxiety.

[41] Bockbrader H.N., Wesche D., Miller R., Chapel S., Janiczek N., Burger P. (2010). A comparison of the pharmacokinetics and pharmacodynamics of pregabalin and gabapentin. Clin. Pharmacokinet..

[42] Bramness J.G., Sandvik P., Engeland A., Skurtveit S. (2010). Does Pregabalin (Lyrica^®^) help patients reduce their use of benzodiazepines? A comparison with gabapentin using the Norwegian Prescription Database. Basic Clin. Pharmacol. Toxicol..

[43] Tomić M., Pecikoza U., Micov A., Vučković S., Stepanović-Petrović R. (2018). Antiepileptic drugs as analgesics/adjuvants in inflammatory pain: Current preclinical evidence. Pharmacol. Ther..

[44] Ranaldi R., Poeggel K. (2002). Baclofen decreases methamphetamine self-administration in rats. Neuroreport.

[45] Marti-Prats L., Belin-Rauscent A., Fouyssac M., Puaud M., Cocker P.J., Everitt B.J., Belin D. (2021). Baclofen decreases compulsive alcohol drinking in rats characterized by reduced levels of GAT-3 in the central amygdala. Addict. Biol..

[46] Kent C.N., Park C., Lindsley C.W. (2020). Classics in Chemical Neuroscience: Baclofen. ACS Chem. Neurosci..

[47] Montoya-Balbás I.J., Valentín-Guevara B., López-Mendoza E., Linzaga-Elizalde I., Ordoñez M., Román-Bravo P. (2015). Efficient Synthesis of β-Aryl-γ-lactams and Their Resolution with (S)-Naproxen: Preparation of (R)- and (S)-Baclofen. Molecules.

[48] Lapin I. (2001). Phenibut (beta-phenyl-GABA): A tranquilizer and nootropic drug. CNS Drug Rev..

[49] Dambrova M., Zvejniece L., Liepinsh E., Cirule H., Zharkova O., Veinberg G., Kalvinsh I. (2008). Comparative pharmacological activity of optical isomers of phenibut. Eur. J. Pharmacol..

[50] Peterson M., Oliveira M. (2004). Preparation of Organic Acid Salts of Gabapentin.

[51] Plata Salaman C.R., Tesson N., Trilla Castano M., Cardenas Romana L. (2011). Crystalline Forms of Pregabalin and Co-Formers in the Treatment of Pain.

[52] Gendron F.-X., Mahieux J., Sanselme M., Coquerel G. (2019). Resolution of Baclofenium Hydrogenomaleate By Using Preferential Crystallization. A First Case of Complete Solid Solution at High Temperature and a Large Miscibility Gap in the Solid State. Cryst. Growth Des..

[53] Báthori N.B., Kilinkissa O.E.Y. (2015). Are gamma amino acids promising tools of crystal engineering?—Multicomponent crystals of baclofen. Cryst. Eng. Comm..

[54] Bloom R.A., Sadrieh N. (2012). Environmental Assessment Finding of No Significant Impact NDA 21-446/S-028 Lyrica (Pregabalin) Capsules. https://www.accessdata.fda.gov/drugsatfda_docs/nda/2012/021446Orig1s028EA.pdf.

[55] Haynes W.M. (2016). CRC Handbook of Chemistry and Physics.

[56] Komisarek D., Haj Hassani Sohi T., Vasylyeva V. (2022). Co-crystals of zwitterionic GABA API’s pregabalin and phenibut: Properties and application. CrystEngComm.

[57] Herbst M., Komisarek D., Strothmann T., Vasylyeva V. (2022). A Lection in Humbleness: Crystallization of Chiral and Zwitterionic APIs Baclofen and Phenibut. Crystals.

[58] Budny-Godlewski K., Leszczyński M.K., Tulewicz A., Justyniak I., Pinkowicz D., Sieklucka B., Kruczała K., Sojka Z., Lewiński J. (2021). A Case Study on the Desired Selectivity in Solid-State Mechano- and Slow-Chemistry, Melt, and Solution Methodologies. ChemSusChem.

[59] Solares-Briones M., Coyote-Dotor G., Páez-Franco J.C., Zermeño-Ortega M.R., de La O Contreras C.M., Canseco-González D., Avila-Sorrosa A., Morales-Morales D., Germán-Acacio J.M. (2021). Mechanochemistry: A Green Approach in the Preparation of Pharmaceutical Cocrystals. Pharmaceutics.

[60] Wang L., Sun G., Zhang K., Yao M., Jin Y., Zhang P., Wu S., Gong J. (2020). Green Mechanochemical Strategy for the Discovery and Selective Preparation of Polymorphs of Active Pharmaceutical Ingredient γ-Aminobutyric Acid (GABA). ACS Sustain. Chem. Eng..

[61] Chikhalia V., Forbes R.T., Storey R.A., Ticehurst M. (2006). The effect of crystal morphology and mill type on milling induced crystal disorder. Eur. J. Pharm. Sci..

[62] Descamps M., Willart J.F. (2016). Perspectives on the amorphisation/milling relationship in pharmaceutical materials. Adv. Drug Deliv. Rev..

[63] Mah P.T., Laaksonen T., Rades T., Aaltonen J., Peltonen L., Strachan C.J. (2014). Unravelling the relationship between degree of disorder and the dissolution behavior of milled glibenclamide. Mol. Pharm..

[64] Kumar S., Prakash O., Gupta A., Singh S. (2019). Solvent-free Methods for Co-crystal Synthesis: A Review. Curr. Org. Synth..

[65] Kulla H., Haferkamp S., Akhmetova I., Röllig M., Maierhofer C., Rademann K., Emmerling F. (2018). In Situ Investigations of Mechanochemical One-Pot Syntheses. Angew. Chem. Int. Ed. Engl..

[66] Muresan-Pop M., Vulpoi A., Simon V., Todea M., Magyari K., Pap Z., Simion A., Filip C., Simon S. (2022). Co-Crystals of Etravirine by Mechanochemical Activation. J. Pharm. Sci..

[67] Beyer M.K., Clausen-Schaumann H. (2005). Mechanochemistry: The mechanical activation of covalent bonds. Chem. Rev..

[68] Agilent (2014). CrysAlisPRO.

[69] Dolomanov O.V., Bourhis L.J., Gildea R.J., Howard J.A.K., Puschmann H. (2009). OLEX2: A complete structure solution, refinement and analysis program. J. Appl. Crystallogr..

[70] Sheldrick G.M. (2008). A short history of SHELX. Acta Crystallogr. A.

[71] Sheldrick G.M. (2015). Crystal structure refinement with SHELXL. Acta Crystallogr. C Struct. Chem..

[72] Macrae C.F., Sovago I., Cottrell S.J., Galek P.T.A., McCabe P., Pidcock E., Platings M., Shields G.P., Stevens J.S., Towler M. (2020). Mercury 4.0: From visualization to analysis, design and prediction. J. Appl. Crystallogr..

[73] Lin M., Tesconi M., Tischler M. (2009). Use of ^1^H NMR to facilitate solubility measurement for drug discovery compounds. Int. J. Pharm..

[74] Zloh M. (2019). NMR spectroscopy in drug discovery and development: Evaluation of physico-chemical properties. ADMET DMPK.

[75] Bharti S.K., Roy R. (2012). Quantitative 1H NMR spectroscopy. TrAC.

[76] Dikmen G. (2018). Determination of the solubility of 2-Methyl-1,3-benzothiazol-5-amine molecule with aqueous ethanol by NMR spectroscopy. J. Mol. Liq..

