# Supplementary material for: Maleic Acid as a Co-Former for Pharmaceutically Active GABA Derivatives: Mechanochemistry or Solvent Crystallization?
Source: Materials (Basel). 2023 Mar 10;16(6):2242. doi: 10.3390/ma16062242 (PMC10054091; doi:10.3390/ma16062242)
Supplement: Supplementary file 1 [file materials-16-02242-s001.zip › materials-2213161-supplementary.pdf]

Supporting Information

1 GABA maleate

Table S1. Single crystal measurement details for GABA maleate.

Parameters	1-MA
Formula	$C_8 H_{13} N O_6$
Formula moiety	$C_4 H_{10} N O_2$, $C_4 H_3 O_4$
M_r [g mol^{-1}]	219.19
Temperature [K]	100(1)
System/space group	monoclinic, $P2_1/c$
a (Å)	5.5757(1)
b (Å)	6.6352 (1)
c (Å)	26.3554(5)
β (°)	94.060(2)
V (Å^3)	972.59(3)
Z/Z’	4/1
Density [g/cm^3]	1.497
μ [mm^{-1}]	1.121
T_{min}/T_{max}	0.93836/ 0.93836
F (000)	464
Crystal size [mm]	0.2 · 0.19 · 0.05
2θ range [°]	3.4 – 67.0
Completeness [%]	100.0
Recorded refl.	9108
Independent refl.	1744
Goodness-of-fit F^2	1.050
X-Ray Source	Cu Kα (λ = 1.54184)
R_1 [%] /wR_2 [%] /S	2.96/ 8.02/ 1.05

GABA maleate was received by either crystallization from aqueous solution or neat grinding of equimolar amounts of GABA and maleic acid (516 mg, 5 mmol and 581 mg, 5 mmol). The same product is received in both cases.

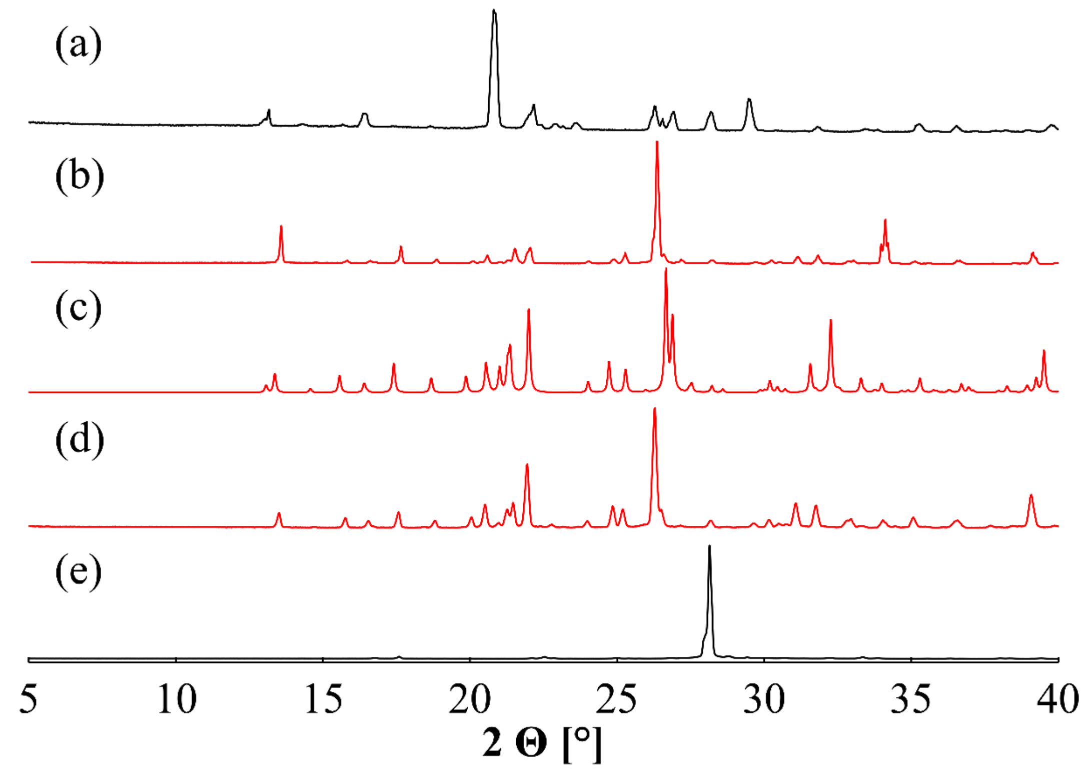

Figure S1. *Powder pattern comparison of (a) recorded GABA pattern, (b) GABA maleate as produced from aqueous solution, (c) GABA maleate pattern simulated by single crystal data, (d) GABA maleate as produced by neat grinding, and (e) recorded maleic acid pattern. A 2Θ range from 5 – 40 ° is depicted.*

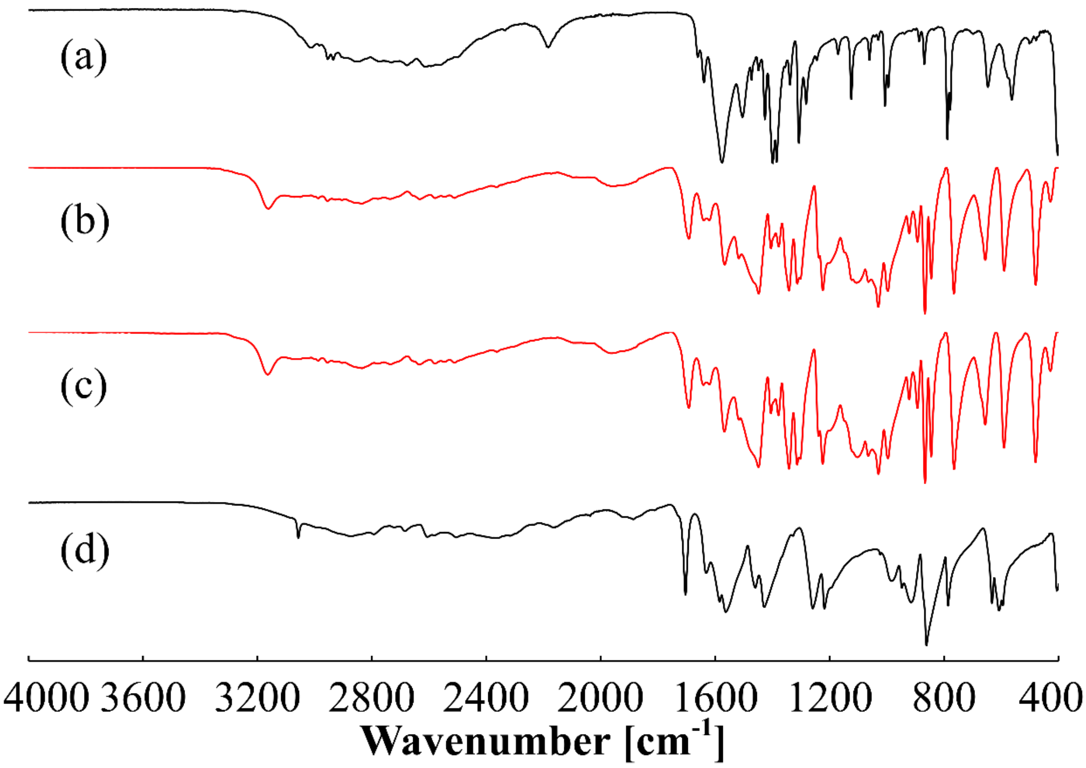

Figure S2. *FT-IR spectra of (a) GABA, (b) GABA maleate as produced from aqueous solution, (c) GABA maleate as produced by neat grinding and (d) maleic acid. Spectra are recorded between 4000 cm^{-1} – 400 cm^{-1}.*

2 Gabapentin maleate hydrate

***Table S2**. Single crystal measurement details for Gabapentin maleate hydrate.*

**Parameters**	**2-MA • H_2O**
**Formula**	$C_{13} H_{23} N O_7$
**Formula moiety**	$C_9 H_{18} N O_2$, $C_4 H_3 O_4$, H_2O
**M_r [g mol^{-1}]**	305.32
**Temperature [K]**	100(1)
**System/space group**	triclinic, $P\bar{1}$
**a (Å)**	6.0214(2)
**b (Å)**	11.7991(6)
**c (Å)**	12.1015(5)
**α (°)**	64.406(4)
**β (°)**	80.251(3)
**γ (°)**	83.629(4)
**V (Å^3)**	763.52(6)
**Z/Z'**	2/1
**Density [g/cm^3]**	1.328
**μ [mm^{-1}]**	0.913
**T_{min}/T_{max}**	0.88604/ 0.88604
**F (000)**	328
**Crystal size [mm]**	0.38 · 0.33 · 0.07
**2θ range [°]**	4.2 – 67.1
**Completeness [%]**	98.8
**Recorded refl.**	7622
**Independent refl.**	2686
**Goodness-of-fit F^2**	1.053
**X-Ray Source**	Cu Kα (λ = 1.54184)
**R_1 [%] /wR_2 [%] /S**	3.71/ 10.27/ 1.05

Gabapentin maleate hydrate was received by crystallization of equimolar amounts of Gabapentin and maleic acid from aqueous solution and an anhydrous form was received via neat grinding of equimolar amounts of Gabapentin and maleic acid (685 mg, 4 mmol and 464 mg, 4 mmol). Both products show clear differences in PXRD patterns as well as FT-IR spectra. Attempts at producing Gabapentin maleate hydrate through liquid-assisted grinding result in a phase mixture of the maleate and the maleate hydrate.

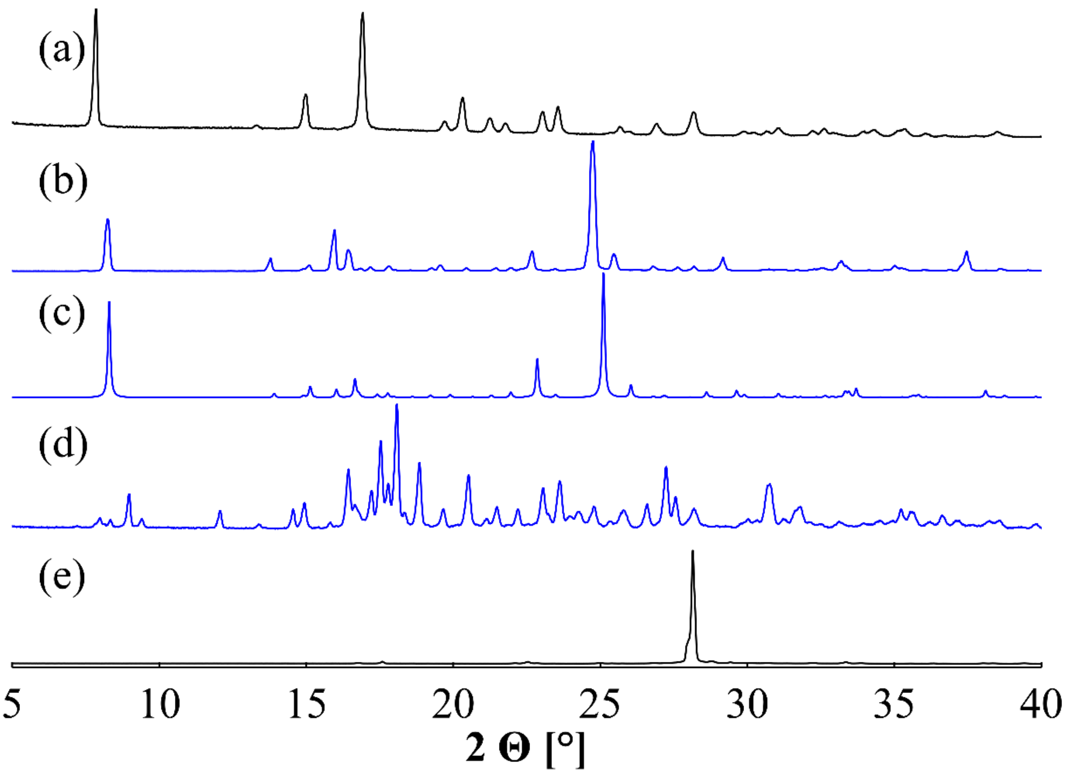

Figure S3. *Powder pattern comparison of (a) recorded Gabapentin pattern, (b) Gabapentin maleate hydrate as produced from aqueous solution, (c) Gabapentin maleate hydrate pattern simulated by single crystal data with hkl = 0 4 0 and March-Dollase parameter of 0.5, (d) Gabapentin maleate as produced by neat-grinding, and (e) recorded Gabapentin pattern. A 2Θ range from 5 – 40 ° is depicted.*

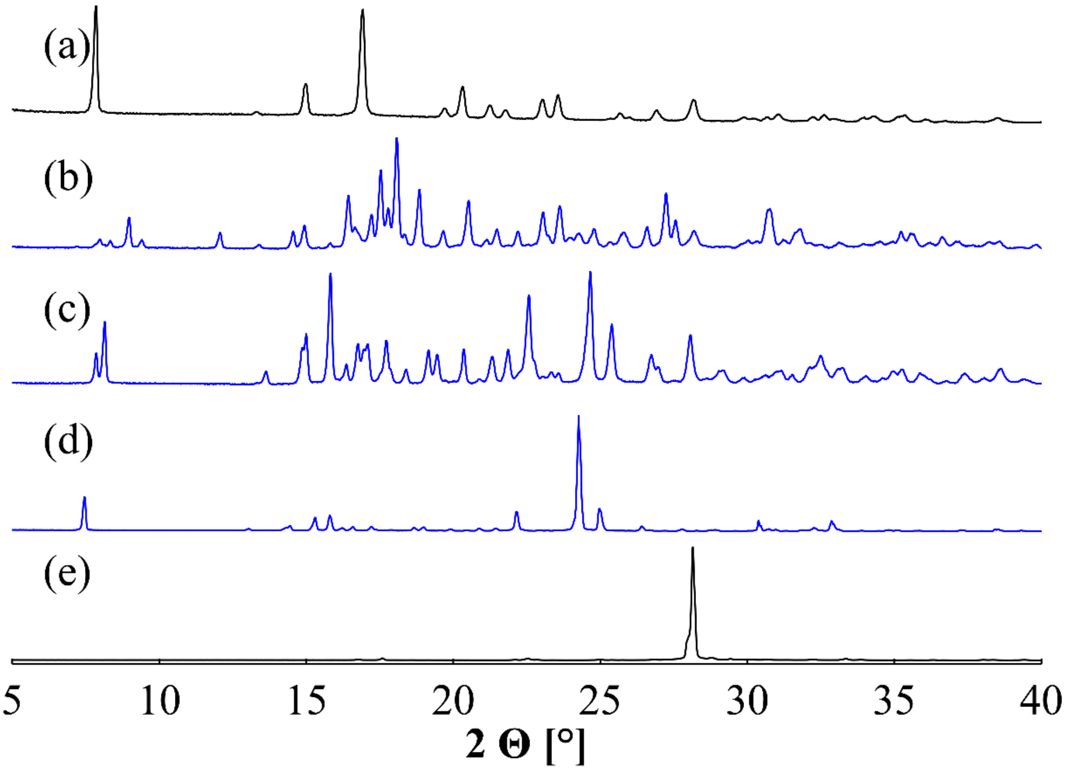

Figure S4*. Powder pattern comparison of (a) recorded Gabapentin pattern, (b) Gabapentin maleate as produced by neat grinding, (c) Gabapentin maleate hydrate and maleate mixture as produced by liquid-assisted grinding, (d) Gabapentin maleate hydrate as produced from aqueous solution and (e) recorded maleic acid pattern. A 2Θ range from 5 – 40 ° is depicted.*

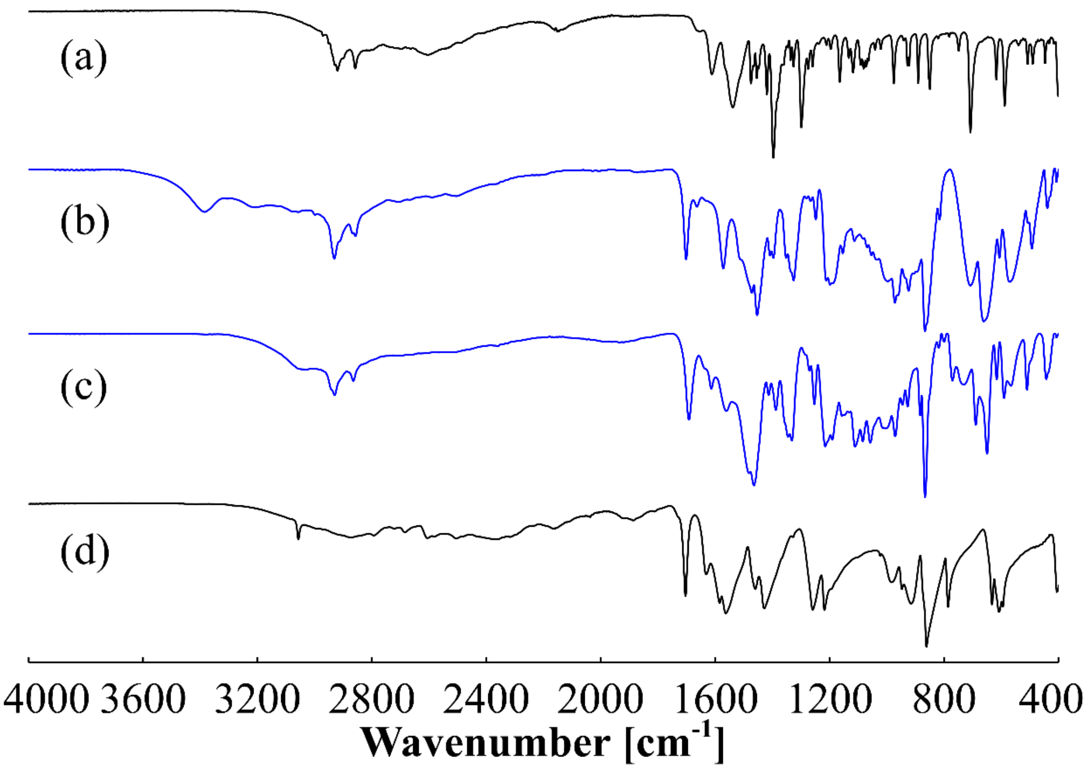

Figure S5. *FT-IR spectra of (a) Gabapentin, (b) Gabapentin maleate hydrate as produced from aqueous solution, (c) Gabapentin maleate as produced by neat grinding and (d) maleic acid. Spectra are recorded between 4000 cm^{-1} – 400 cm^{-1}.*

3 (*rac*)-Pregabalin maleate

Table S3. *Single crystal measurement details for (rac)-Pregabalin maleate.*

**Parameters**	**(*rac*)-3-MA-I**
**Formula**	$C_{12} H_{21} N O_6$
**Formula moiety**	$C_8 H_{18} N O_2$, $C_4 H_3 N O_4$
**M_r [g mol^{-1}]**	275.30
**Temperature [K]**	100(1)
**System/space group**	triclinic, $P\bar{1}$
**a (Å)**	5.8681(2)
**b (Å)**	11.1369(3)
**c (Å)**	11.2720(3)
**α (°)**	88.527(2)
**β (°)**	79.402(2)
**γ (°)**	75.521(2)
**V (Å^3)**	700.93(4)
**Z/Z'**	2/1
**Density [g/cm^3]**	1.304
**μ [mm^{-1}]**	0.882
**T_{min}/T_{max}**	0.97036/ 0.97036
**F (000)**	296
**Crystal size [mm]**	0.22 · 0.11 · 0.02
**2θ range [°]**	4.0 – 67.1
**Completeness [%]**	99.5
**Recorded refl.**	16382
**Independent refl.**	2484
**Goodness-of-fit F^2**	1.075
**X-Ray Source**	Cu Kα (λ = 1.54184)
**R_1 [%] /wR_2 [%] /S**	4.23/ 11.35/ 1.08

(*rac*)-Pregabalin maleate mixtures of form I and II were received by either crystallization from aqueous solution or neat grinding of equimolar amounts of (*rac*)-Pregabalin hydrate and maleic acid (637 mg, 4 mmol and 464 mg, 4 mmol). The same product is received in both cases, though in one grinding experiment the content of form II was higher. IR-spectra highlight phase shifts between the two forms.

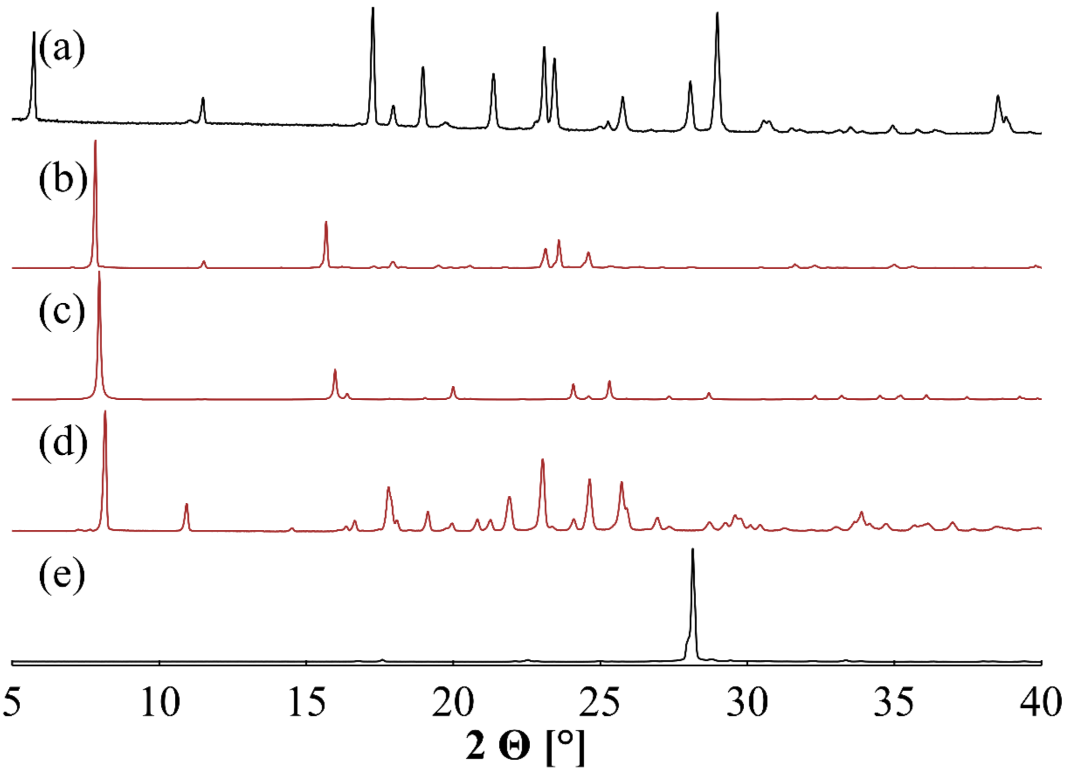

Figure S6. *Powder pattern comparison of (a) recorded (rac)-Pregabalin pattern, (b) (rac)-Pregabalin maleate as produced from aqueous solution, (c) (rac)-Pregabalin maleate I pattern simulated by single crystal data with hkl = 4 15 2 and March-Dollase parameter of 4, (d) (rac)-Pregabalin maleate pattern produced via neat grinding with higher content of II, and (e) recorded maleic acid pattern. A 2Θ range from 5 – 40 ° is depicted.*

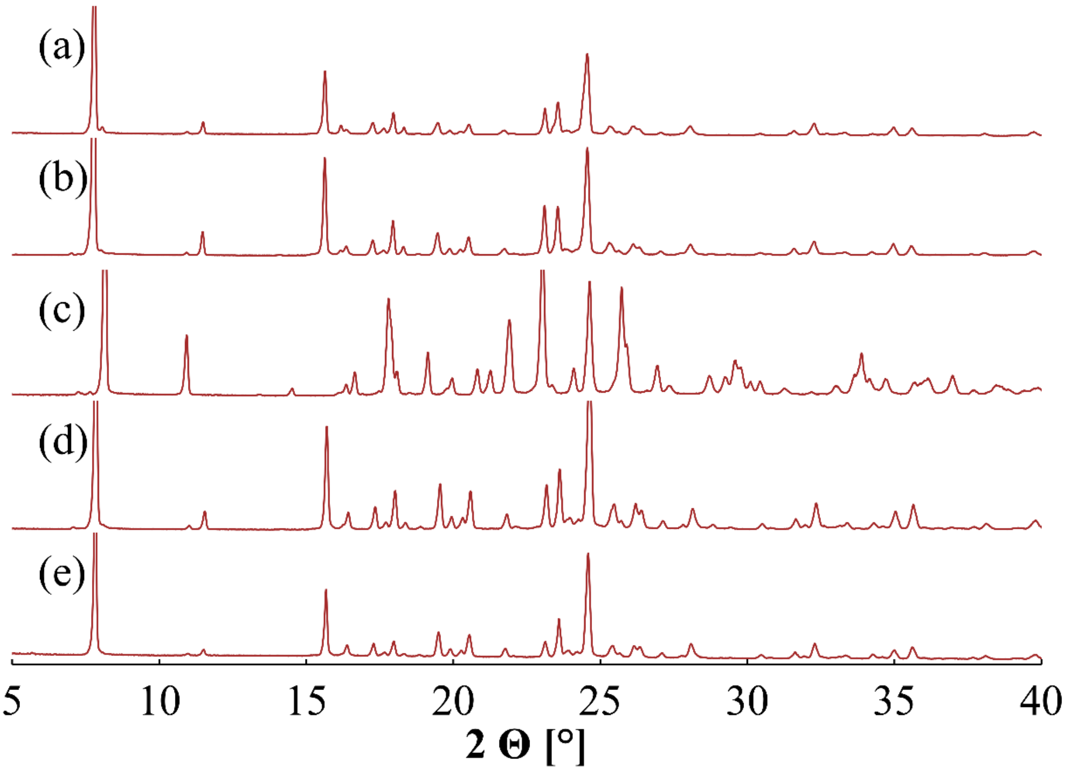

Figure S7. *Various recorded powder patterns of solvent grown (a), (b) as well as mechanochemically prepared (d), (e) (rac)-Pregabalin maleates compared to the once received phase containing more of form II (c).*

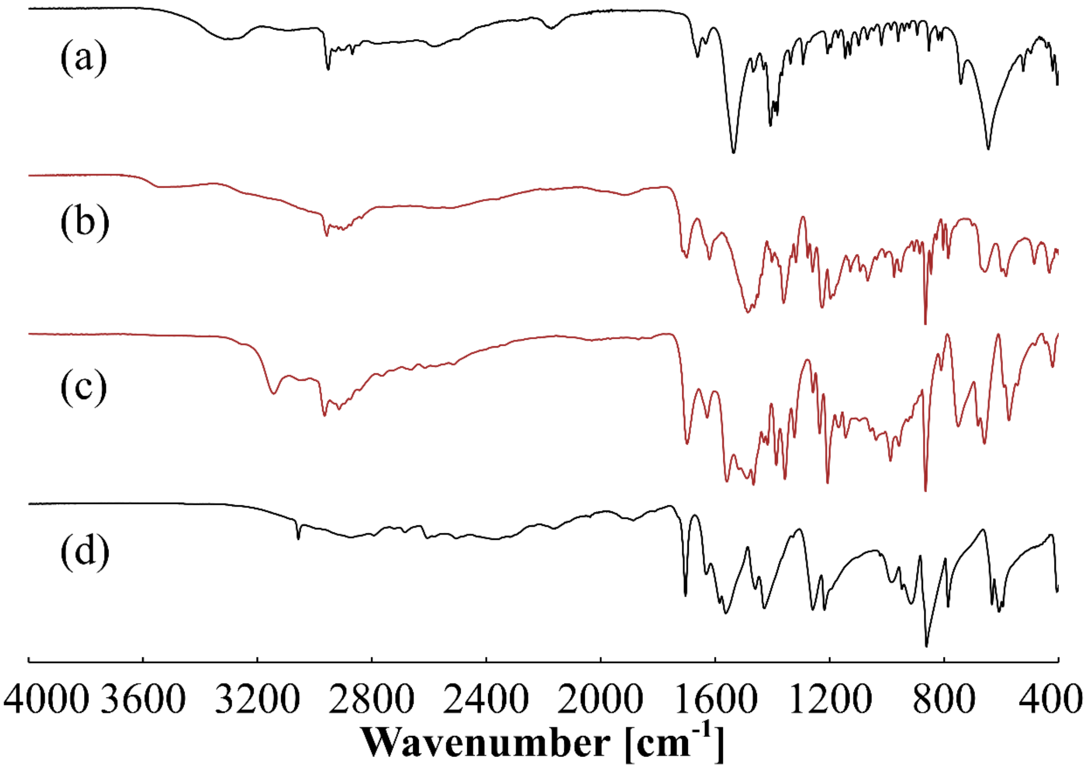

Figure S8. *FT-IR spectra of (a) (rac)-Pregabalin hydrate, (b) (rac)-Pregabalin maleate as produced from aqueous solution, (c) (rac)-Pregabalin maleate as produced by neat grinding containing more of form II and (d) maleic acid. Spectra are recorded between 4000 cm^{-1} – 400 cm^{-1}.*

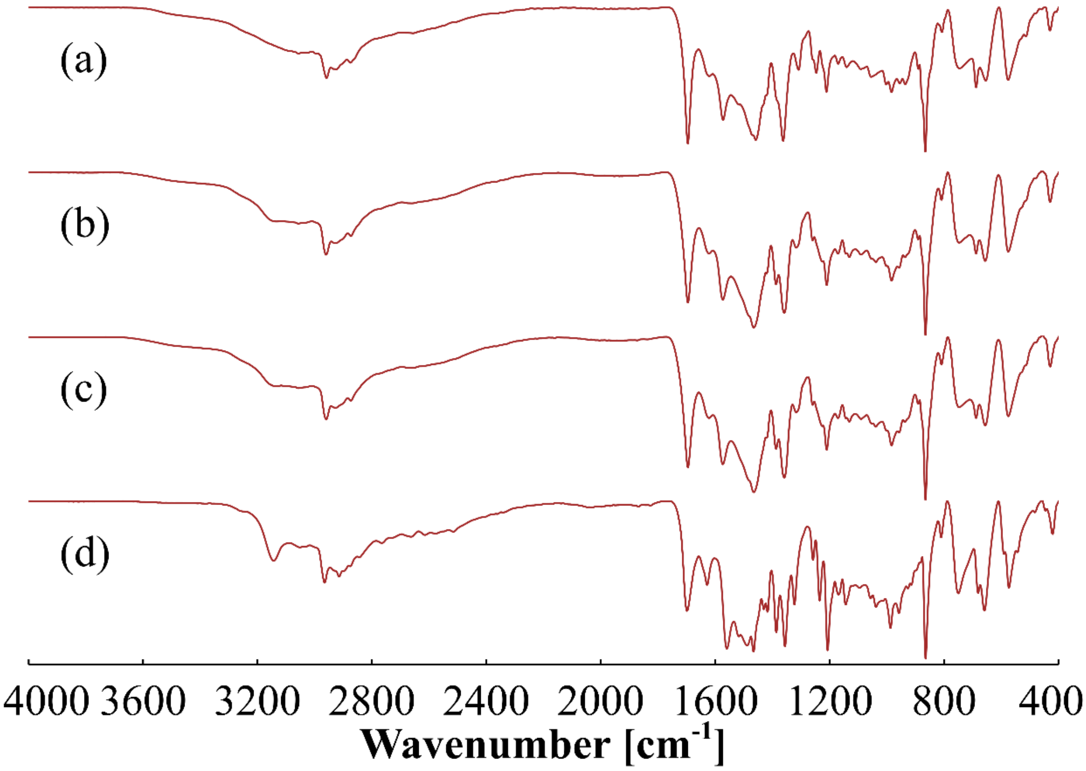

Figure S9. *FT-IR spectra of (a) (rac)-Pregabalin maleate as produced from aqueous solution after three days drying, (b) (rac)-Pregabalin maleate as produced from aqueous solution, two weeks drying, (c) (rac)-Pregabalin maleate as produced from aqueous solution, two weeks drying and subsequent vacuum drying at 40 °C for 2 h, and (d) (rac)-Pregabalin maleate as produced by neat grinding containing more of form II. Spectra are recorded between 4000 cm^{-1} – 400 cm^{-1}.*

4 (*S*)-Pregabalin maleate • H_2O

Table S4. *Single crystal measurement details for (S)-Pregabalin maleate hydrate.*

**Parameters**	**(*S*)-3-MA • H_2O**
**Formula**	$C_{24} H_{44} N_2 O_{13}$
**Formula moiety**	$2(C_8 H_{18} N O_2)$, $2(C_4 H_3 O_4)$, $2(H_2O$ * 0.5)
**M_r [g mol^{-1}]**	568.61
**Temperature [K]**	100(1)
**System/space group**	monoclinic, *C*2
**a (Å)**	29.9006(3)
**b (Å)**	5.5788(1)
**c (Å)**	18.0925(2)
**β (°)**	102.615(1)
**V (Å^3)**	2945.14(7)
**Z/Z'**	4/2
**Density [g/cm^3]**	1.282
**μ [mm^{-1}]**	0.881
**T_{min}/T_{max}**	0.629/ 1.000
**F (000)**	1224
**Crystal size [mm]**	0.52 · 0.10 · 0.06
**2θ range [°]**	2.5 – 67.1
**Completeness [%]**	99.9
**Recorded refl.**	44205
**Independent refl.**	5271
**Flack x**	0.01(3)
**Goodness-of-fit F^2**	1.033
**X-Ray Source**	Cu Kα (λ = 1.54184)
**R_1 [%] /wR_2 [%] /S**	3.50/ 9.02/ 1.03

(S)-Pregabalin maleate hydrate was received by either crystallization from aqueous solution or liquid-assisted grinding of equimolar amounts of (S)-Pregabalin and maleic acid (477 mg 3 mmol and 384 mg 3 mmol). The same product is received in both cases, though the presence of water is visible in FT-IR spectra of samples grown through solvent evaporation. Attempts at producing (S)-Pregabalin maleate hydrate through neat grinding result in lower crystallinity and an incomplete conversion of the co-formers. The solvent product can be dried at 40 °C under vacuum conditions of 10^{-3} bar.

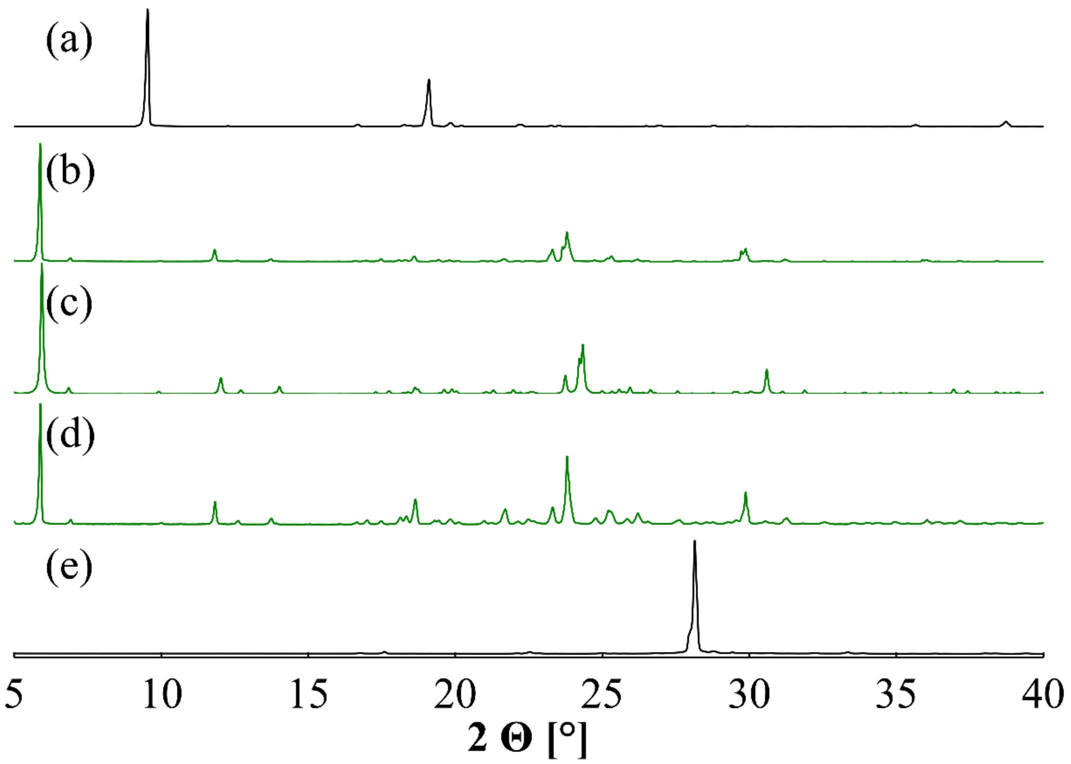

Figure S10. Powder pattern comparison of (a) recorded (S)-Pregabalin pattern, (b) (S)-Pregabalin maleate hydrate as produced from aqueous solution, (c) (S)-Pregabalin maleate hydrate pattern simulated by single crystal data with hkl = 0 1 1 and March-Dollase parameter of 2, (d) (S)-Pregabalin maleate hydrate as produced by liquid-assisted grinding and (e) recorded maleic acid pattern. A 2Θ range from 5 – 40 ° is depicted.

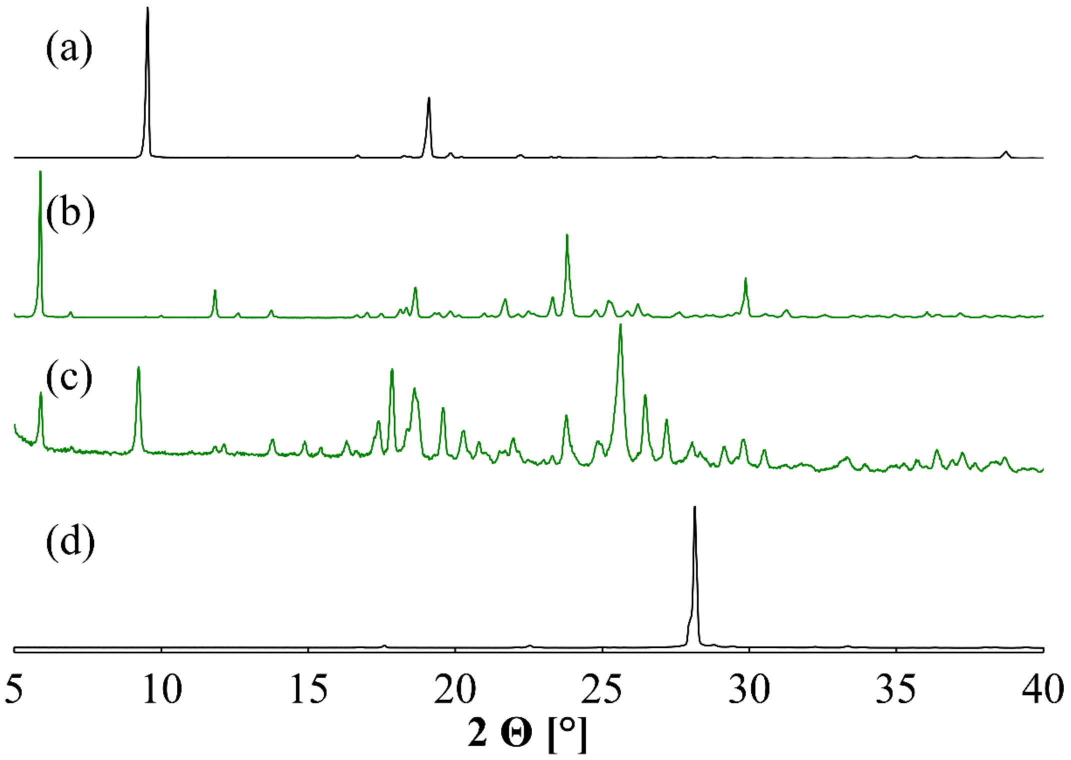

Figure S11. Powder pattern comparison of (a) recorded (S)-Pregabalin pattern, (b) (S)-Pregabalin maleate hydrate as produced by liquid-assisted grinding, (c) (S)-Pregabalin maleate pattern produced by neat grinding and (d) recorded maleic acid pattern. A 2Θ range from 5 – 40 ° is depicted.

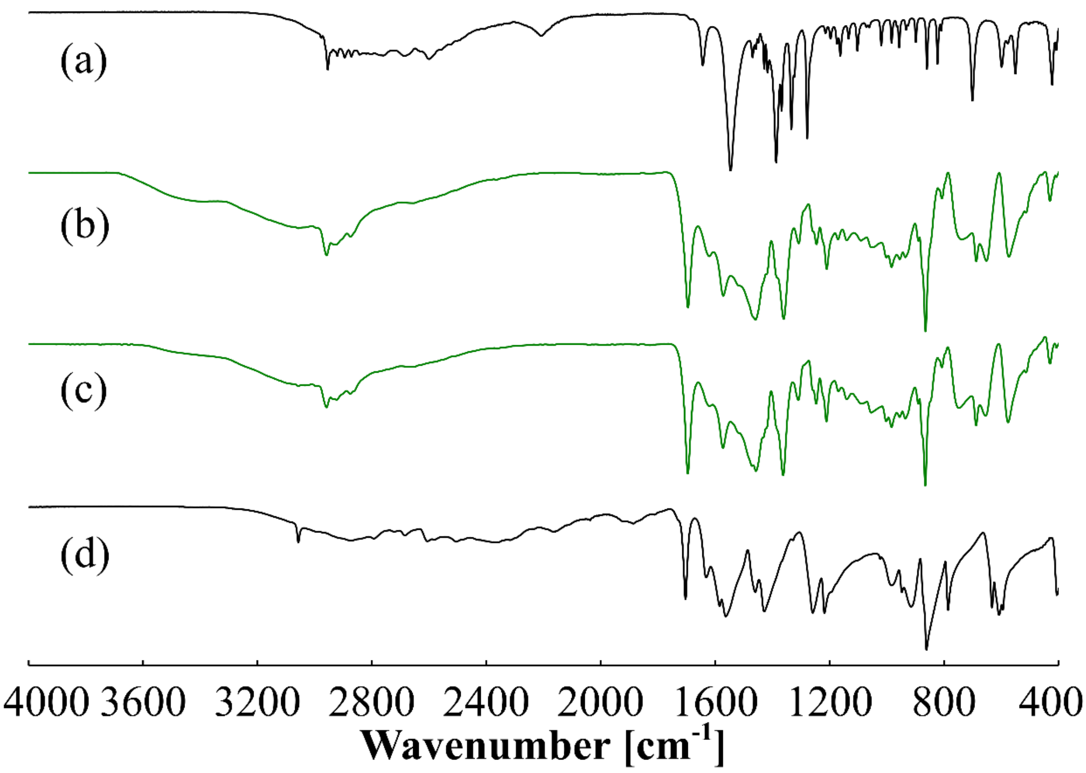

Figure S12. *FT-IR spectra of (a) (S)-Pregabalin, (b) (S)-Pregabalin maleate hydrate as produced from aqueous solution, (c) (S)-Pregabalin maleate hydrate as produced by liquid-assisted grinding and (d) maleic acid. Spectra are recorded between 4000 cm^{-1} – 400 cm^{-1}.*

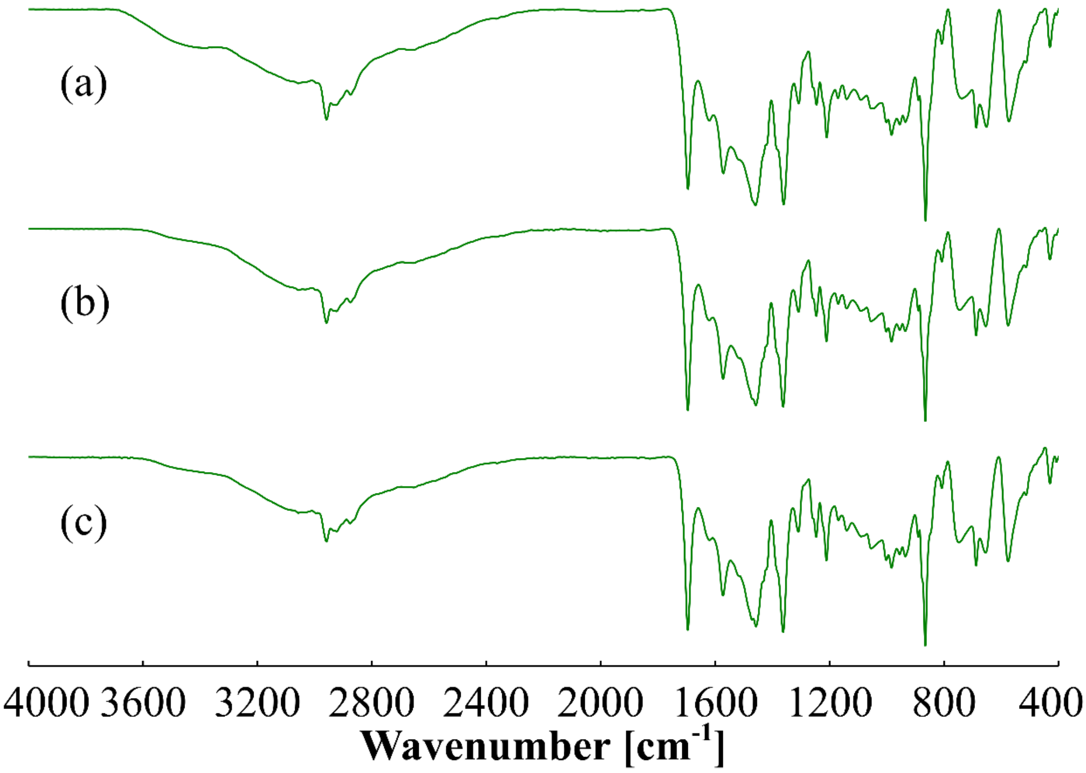

Figure S13*. FT-IR spectra of (a) (S)-Pregabalin maleate hydrate as produced from aqueous solution, three days drying, (b) (S)-Pregabalin maleate as produced from aqueous solution, vacuum drying and (c) (S)-Pregabalin maleate hydrate as produced by liquid-assisted grinding. Spectra are recorded between 4000 cm^{-1} – 400 cm^{-1}.*

5 Phenibut maleate

Table S5. Single crystal measurement details for Phenibut maleate.

Parameters	4-MA
**Formula**	$C_{14} H_{17} N O_6$
**Formula moiety**	$C_{10} H_{14} N O_2$, $C_4 H_3 O_4$
**M_r [g mol^{-1}]**	295.28
**Temperature [K]**	100(1)
**System/space group**	monoclinic, *P*21/*c*
**a (Å)**	5.7053(1)
**b (Å)**	36.4004(5)
**c (Å)**	7.2941(2)
**β (°)**	112.200(2)
**V (Å^3)**	1402.51(5)
**Z/Z'**	4/1
**Density [g/cm^3]**	1.398
**μ [mm^{-1}]**	0.932
**T_{min}/T_{max}**	0.77073/ 1.00000
**F (000)**	624
**Crystal size [mm]**	0.21 · 0.17 · 0.08
**2θ range [°]**	2.4 – 77.9
**Completeness [%]**	98.9
**Recorded refl.**	15078
**Independent refl.**	2748
**Goodness-of-fit F^2**	1.117
**X-Ray Source**	Cu Kα (λ = 1.54184)
**R_1 [%] /wR_2 [%] /S**	4.22/ 10.10/ 1.12

Phenibut maleate was received by either crystallization from aqueous solution or liquid-assisted grinding of equimolar amounts of Phenibut and maleic acid (717 mg, 4 mmol and 464 mg, 4 mmol). Both received phases must be considered impure as they do not fit the powder pattern simulated by single crystal data.

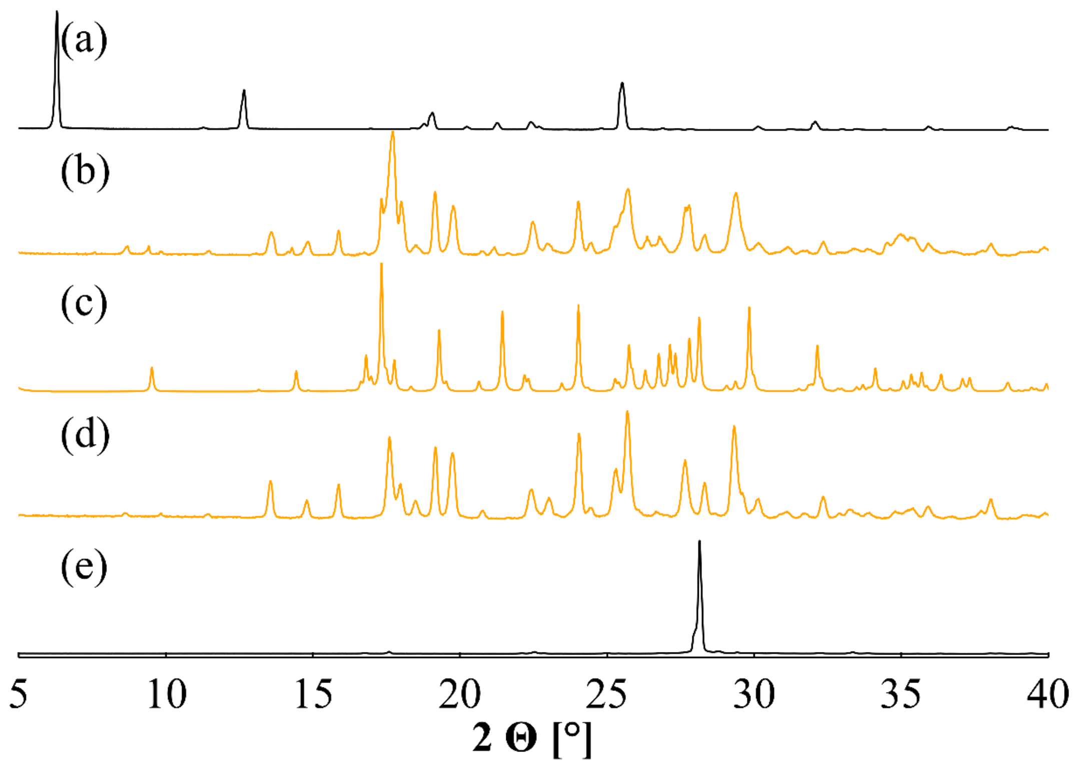

Figure S14. *Powder pattern comparison of (a) recorded Phenibut pattern, (b) Phenibut maleate as produced from aqueous solution, (c) Phenibut maleate pattern simulated by single crystal data with hkl = 1 8 1 and March-Dollase parameter of 0.65, (d) Phenibut maleate as produced by neat grinding and (e) recorded maleic acid pattern. A 2Θ range from 5 – 40 ° is depicted.*

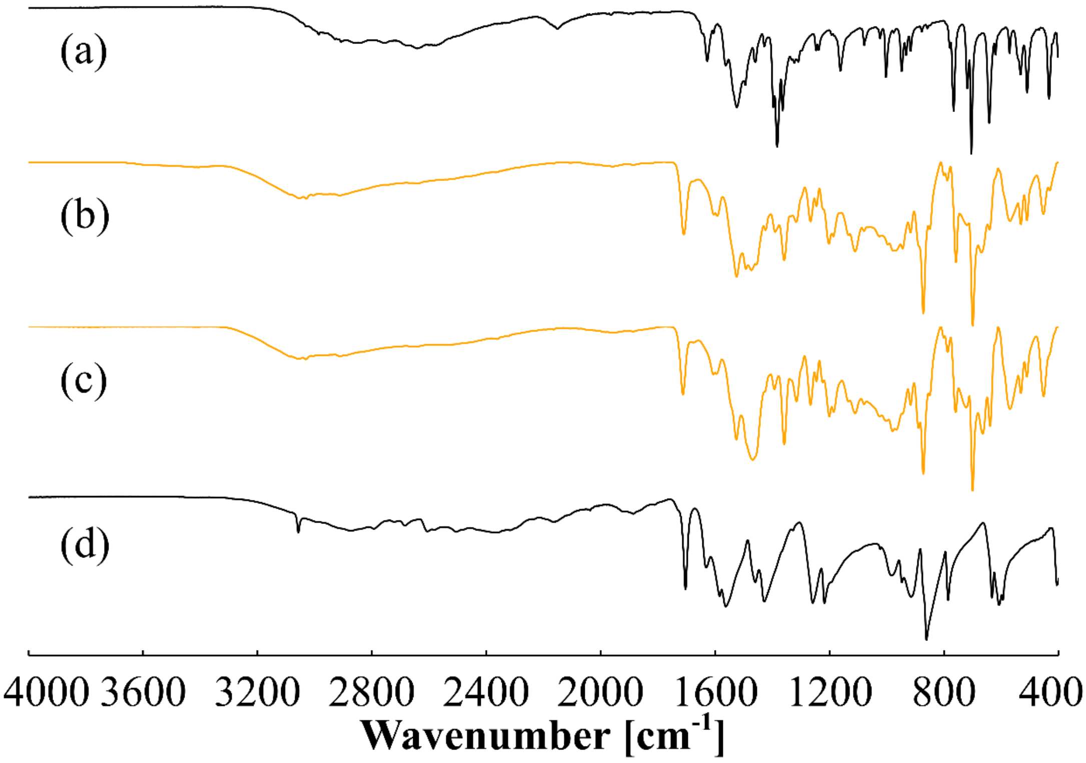

Figure S15. *FT-IR spectra of (a) Phenibut, (b) Phenibut maleate as produced from aqueous solution, (c) Phenibut maleate as produced by neat grinding and (d) maleic acid. Spectra are recorded between 4000 cm^{-1} – 400 cm^{-1}.*

6 Baclofen maleate

Table S6. *Single crystal measurement details for Baclofen maleate.*

**Parameters**	**5-MA**
**Formula**	$C_{14} H_{16} Cl N O_6$
**Formula moiety**	$C_{10} H_{13} Cl N O_2$, $C_4 H_3 O_4$
**M_r [g mol^{-1}]**	329.73
**Temperature [K]**	100(1)
**System/space group**	monoclinic, *P*21
**a (Å)**	5.7000(1)
**b (Å)**	13.5881(2)
**c (Å)**	9.6176(1)
**β (°)**	106.886(1)
**V (Å^3)**	712.787(18)
**Z/Z'**	2/1
**Density [g/cm^3]**	1.536
**μ [mm^{-1}]**	2.667
**T_{min}/T_{max}**	0.96014/ 0.96014
**F (000)**	344
**Crystal size [mm]**	0.21 · 0.11 · 0.06
**2θ range [°]**	4.8 – 67.1
**Completeness [%]**	99.8
**Recorded refl.**	7998
**Independent refl.**	2279
**Goodness-of-fit F^2**	1.093
**X-Ray Source**	Cu Kα (λ = 1.54184)
**R_1 [%] /wR_2 [%] /S**	2.71/ 6.85/ 1.09

Baclofen maleate was received by either crystallization from aqueous solution or neat grinding of equimolar amounts of Baclofen and maleic acid (641 mg, 3 mmol and 348 mg, 3 mmol). The same product is received in both cases, though the crystallinity is lower in the milling product of Baclofen maleate.

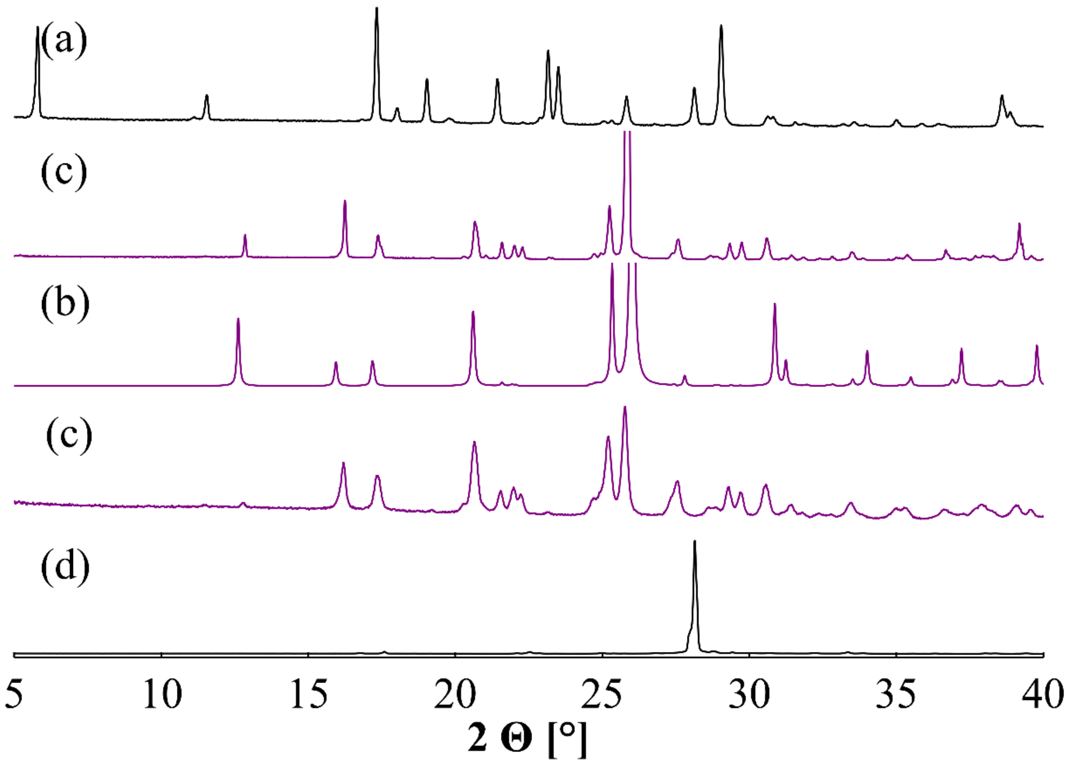

Figure S16. *Powder pattern comparison of (a) recorded Baclofen pattern, (b) Baclofen maleate as produced from aqueous solution, (c) Baclofen maleate pattern simulated by single crystal data with hkl = 0 0 7 and March-Dollase parameter of 4, (d) Baclofen maleate as produced by neat grinding and (e) recorded maleic acid pattern. A 2Θ range from 5 – 40 ° is depicted.*

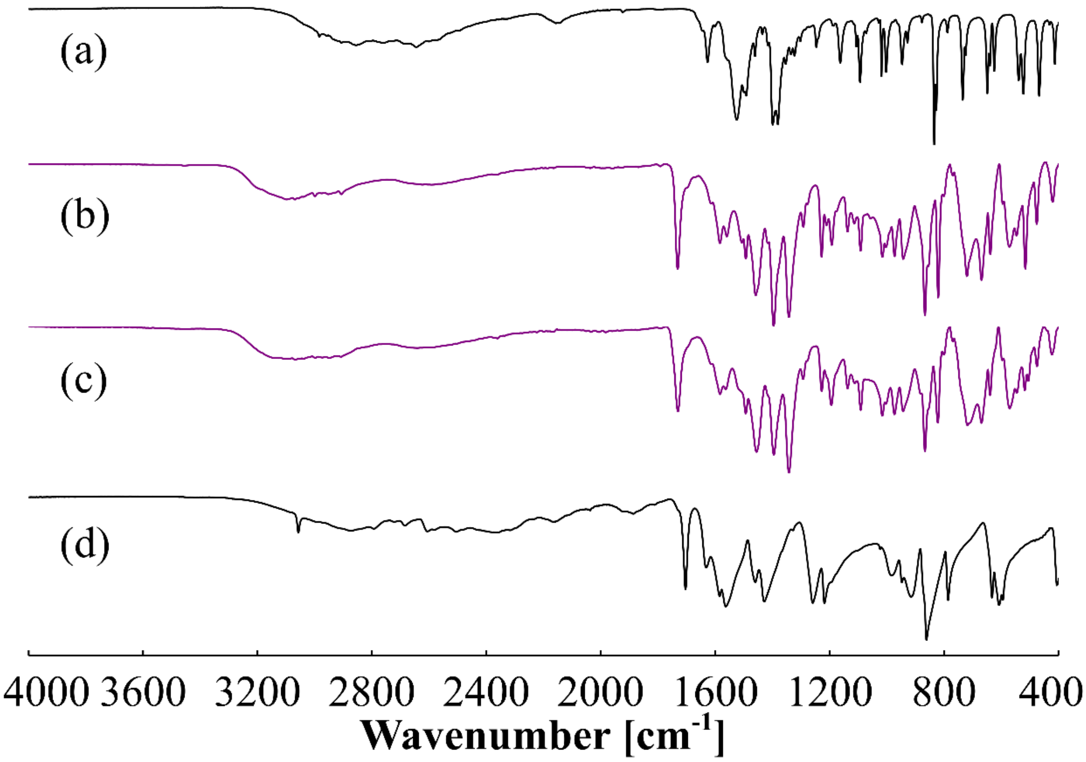

Figure S17. *FT-IR spectra of (a) Baclofen, (b) Baclofen maleate as produced from aqueous solution, (c) Baclofen maleate as produced by neat grinding and (d) maleic acid. Spectra are recorded between 4000 cm^{-1} – 400 cm^{-1}.*

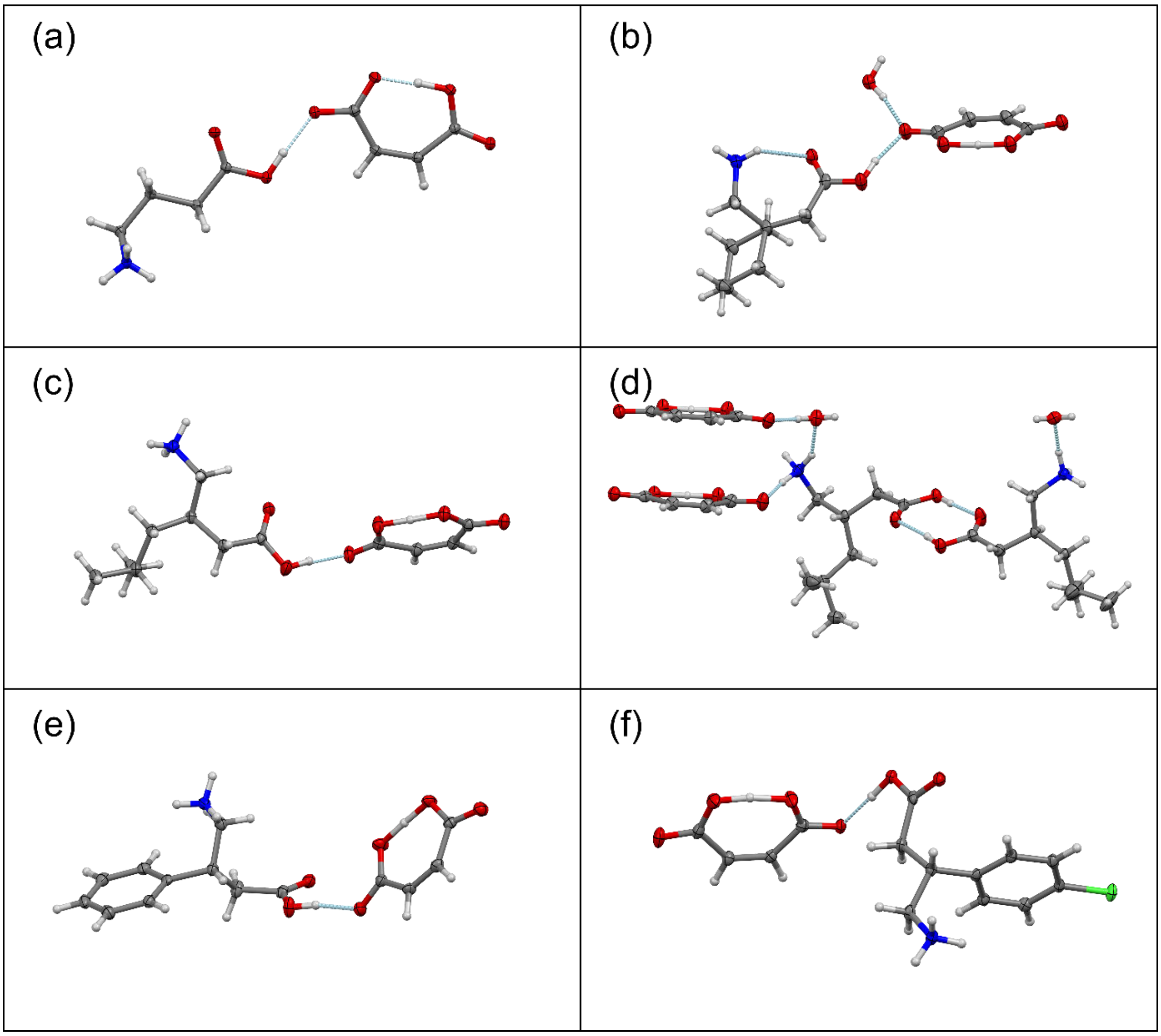

Figure S18. *Depiction of the asymmetric unit in each compound that could be characterized by SCXRD: (a) GABA maleate, (b) Gabapentin maleate hydrate, (c) (rac)-Pregabalin maleate, (d) (S)-Pregabalin maleate hydrate, (e) Phenibut maleate, and (f) Baclofen maleate. Carbon atoms are depicted in grey, hydrogen atoms in white, nitrogen atoms in blue, oxygen atoms in red, and chlorine atoms in green. Hydrogen bonds occurring in the asymmetric units are shown as light blue dotted lines.*

7 Solubility determination details

Solubilities were determined with ^{1}H-NMR. Three samples for each single compound and three samples for each received maleate gained through grinding or solvent crystallization were prepared. For this, dispersions of the investigated compounds in water were prepared in such a way that no complete dissolution would occur after three days at 25 °C in an incubator shaking with 60 min^{-1}. Subsequently, 50 µL of liquid were removed from the samples and added to 450 µL of D_2O prepared in an NMR-tube. ^{1}H-NMR measurements were conducted on a Bruker Avance III NMR-spectrometer at 600 MHz. Solubilities were determined by phase and baseline correction of the received spectrum in MestReNova software x64 14.2.0, application of D_2O shift on the solvent peak from the MestReNova

database. Integration of a chosen signal in the received spectrum and further integration of the solvent signal. The latter signal was always integrated in the borders of 5.000 – 4.600 ppm, the product signals were integrated in as narrow a range as possible (**Table S7**). Trace water in D_2O was considered negligible. Solubilities were calculated by the following equation:

$$S_p = \left(\frac{M_p}{\frac{I_w}{n} M_w} \rho_w \right) \tag{1}$$

Where S_p is the solubility of the API in gL^{-1}, M_p is the molar mass of the targeted API in g mol^{-1}, I_w is the value of the water integral received, n is an adjustment factor necessary if the product integration value is not one (if integral product = 1, n = 1, if integral product > 1, n = 2 because solvent is water), M_w is the molar mass of water in $gmol^{-1}$ and ρ_w is the density of water at 25 °C in gL^{-1}. Error was calculated as the standard deviation of the three recorded samples according to **Equation 2**.

$$V = \sqrt{\frac{\sum(x - \bar{x})^2}{(n-1)}} \tag{2}$$

Here V is the standard deviation, x is the average of the three solubility values, $\bar{x}$ each single solubility value and n the number of samples, three.

Table S7. *Solubilities of GABA and its derivatives on their own and in form of the investigated maleates and their standard deviations. The integral borders for the product peaks used for solubility calculations of samples S1 – S3 are given.*

Sample	$S_p \pm V$ [gL^{-1}]	Int. S1[ppm]	Int. S2 [ppm]	Int. S3[ppm]
**MA**	687 ± 44	6.380 – 6.300	6.380 – 6.300	6.380 – 6.300
**1**	2261 ± 22	1.875 – 1.800	1.875 – 1.800	1.875 – 1.800
**2**	174 ± 7	2.440 – 2.410	2.440 – 2.410	2.440 – 2.410
**(*rac*)-3 • H_2O**	33 ± 1	0.915 – 0.870	0.925 – 0.880	0.915 – 0.870
**(*S*)-3**	41 ± 1	0.915 – 0.870	0.915 – 0.870	0.920 – 0.875
**4**	15 ± 0.3	7.475 – 7.340	7.475 – 7.340	7.475 – 7.340
**5**	3 ± 0.1	7.475 – 7.340	7.475 – 7.340	7.475 – 7.340
**1-MA_sol**	704 ± 60	1.930 – 1.850	1.930 – 1.850	1.920 – 1.840
**1-MA_mill**	680 ± 46	1.930 – 1.850	1.930 – 1.850	1.925 – 1.845
**2-MA • H_2O_sol**	241 ± 8	2.530 – 2.500	2.530 – 2.500	2.530 – 2.500
**2-MA_mill**	218 ± 8	2.530 – 2.500	2.530 – 2.500	2.530 – 2.500
**(*rac*)-3-MA_sol**	719 ± 10	0.870 – 0.815	0.870 – 0.815	0.870 – 0.815
**(*rac*)-3-MA_mill**	559 ± 19	0.880 – 0.800	0.880 – 0.800	0.880 – 0.800
**(*S*)-3-MA• H_2O_sol**	977 ± 79	0.860 – 0.805	0.860 – 0.805	0.860 – 0.805
**(*S*)-3-MA• H_2O_mill**	809 ± 8	0.860 – 0.805	0.860 – 0.805	0.860 – 0.805
**4-MA_sol**	124 ± 4	7.475 – 7.340	7.475 – 7.340	7.475 – 7.340
**4-MA_mill**	128 ± 8	7.475 – 7.340	7.475 – 7.340	7.475 – 7.340
**5-MA_sol**	6 ± 0.3	7.475 – 7.340	7.475 – 7.340	7.475 – 7.340
**5-MA_mill**	6 ± 0.6	7.480 – 7.330	7.480 – 7.330	7.480 – 7.330

Chosen ^{1}H-NMR spectra recorded for these solubility measurements are shown in **Figures S19 – S32**.

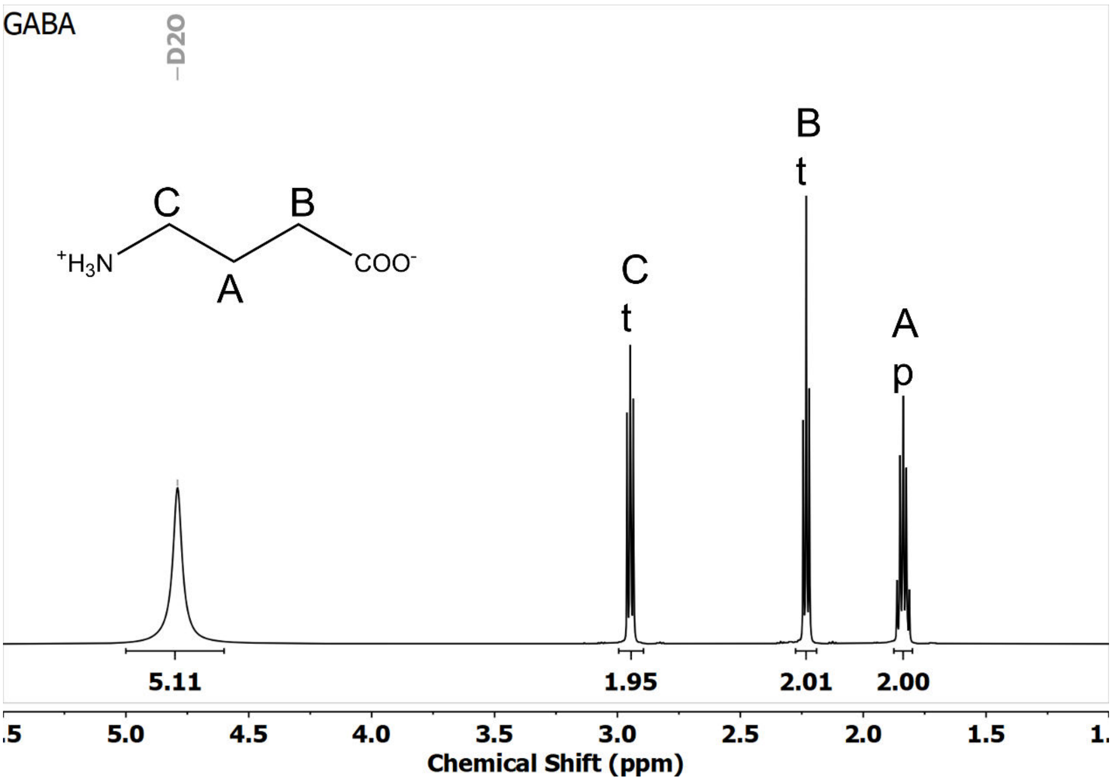

Figure S19. ^{1}H-NMR spectrum of GABA recorded in D_2O at 600 MHz.

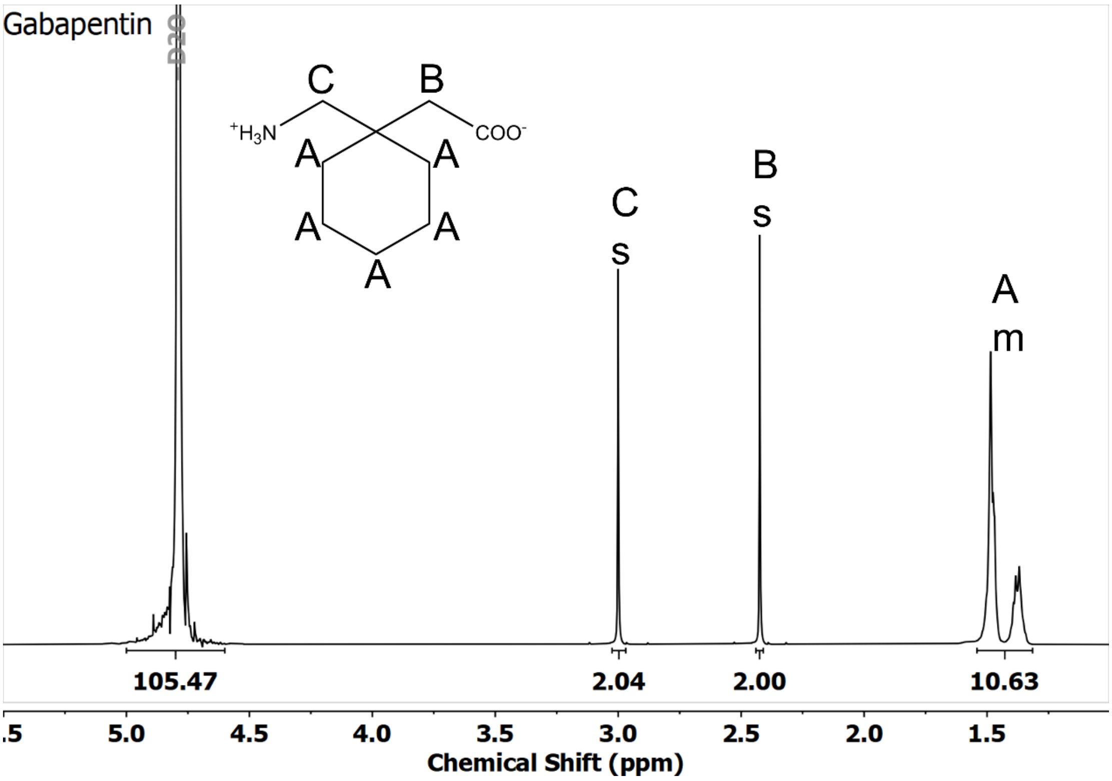

Figure S20. ^{1}H-NMR spectrum of Gabapentin recorded in D_2O at 600 MHz.

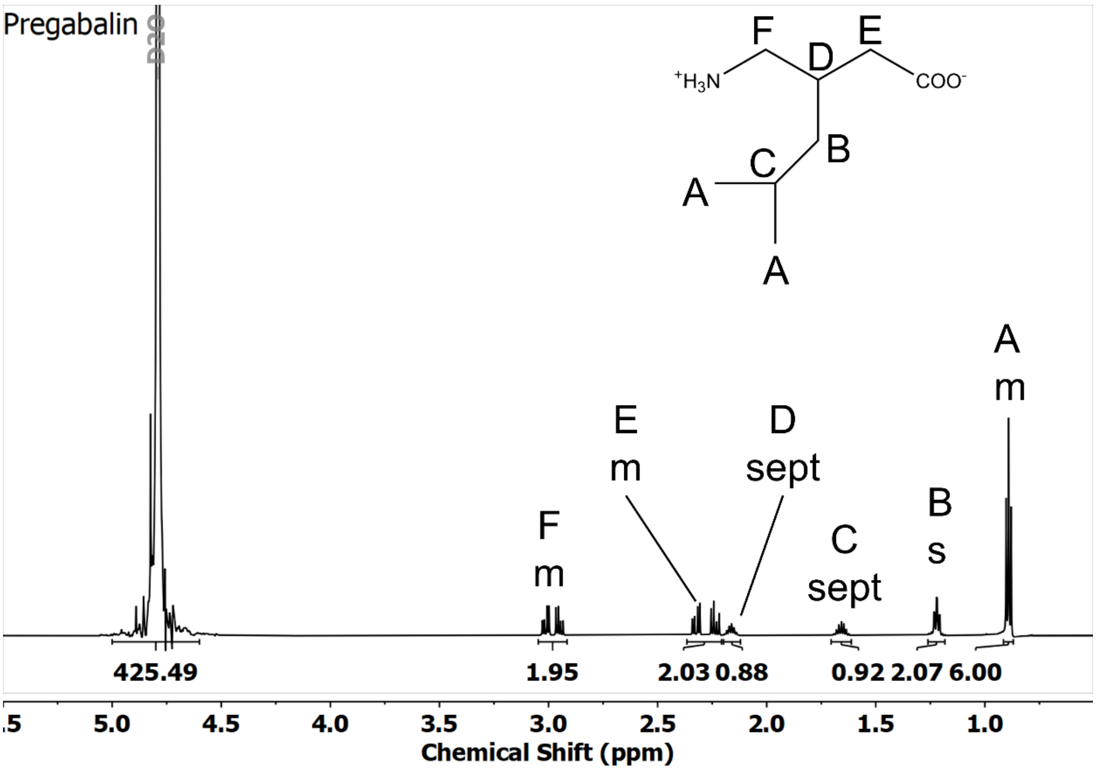

Figure S21. *^{1}H-NMR spectrum of (S)-Pregabalin recorded in D_2O at 600 MHz. Also represents (rac)-Pregabalin.*

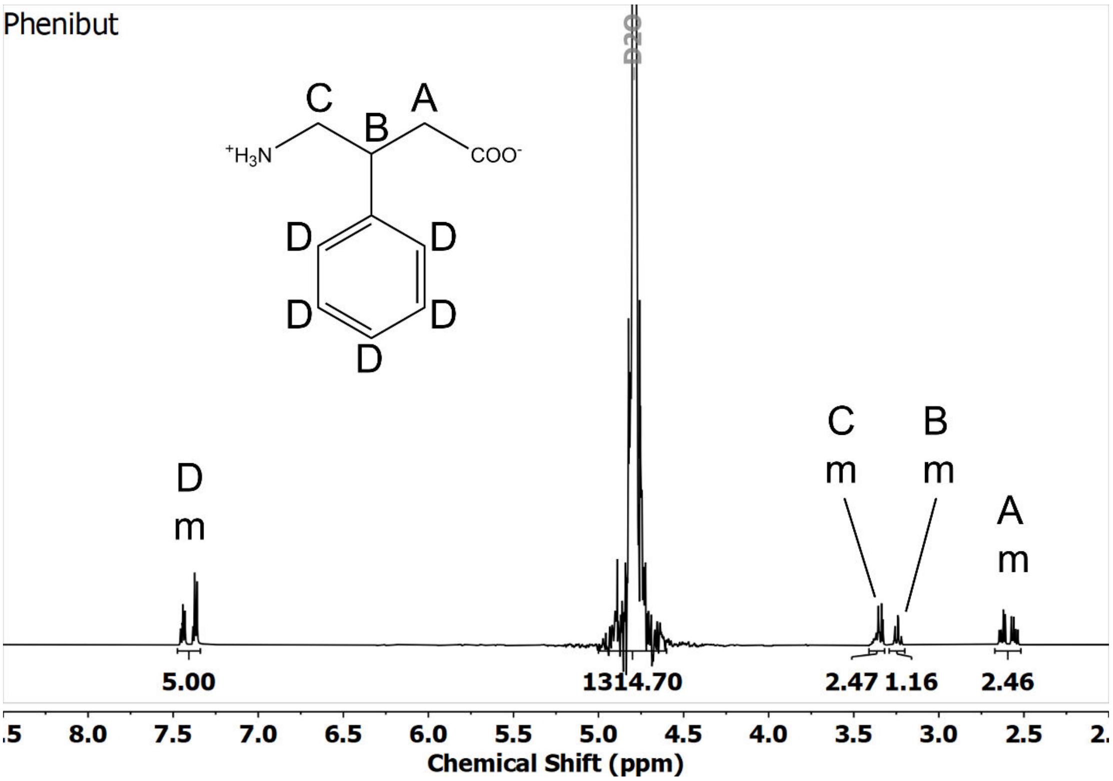

Figure S22. *^{1}H-NMR spectrum of Phenibut recorded in D_2O at 600 MHz.*

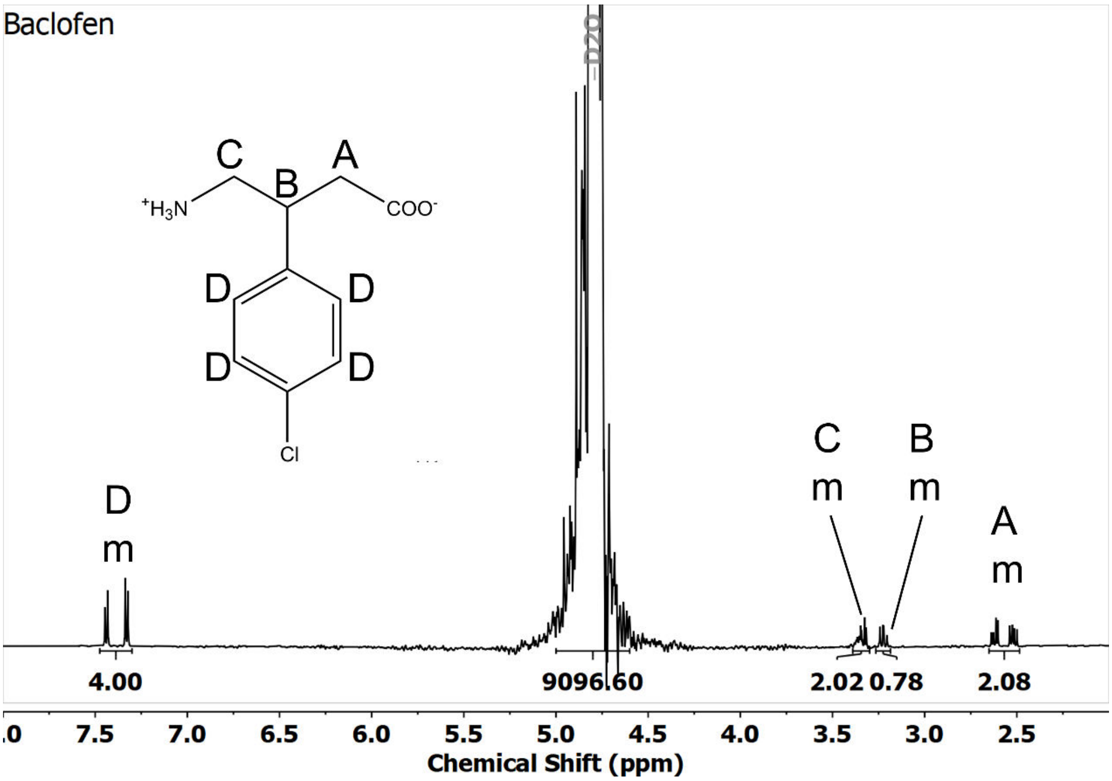

Figure S23. *^{1}H-NMR spectrum of Baclofen recorded in D_2O at 600 MHz.*

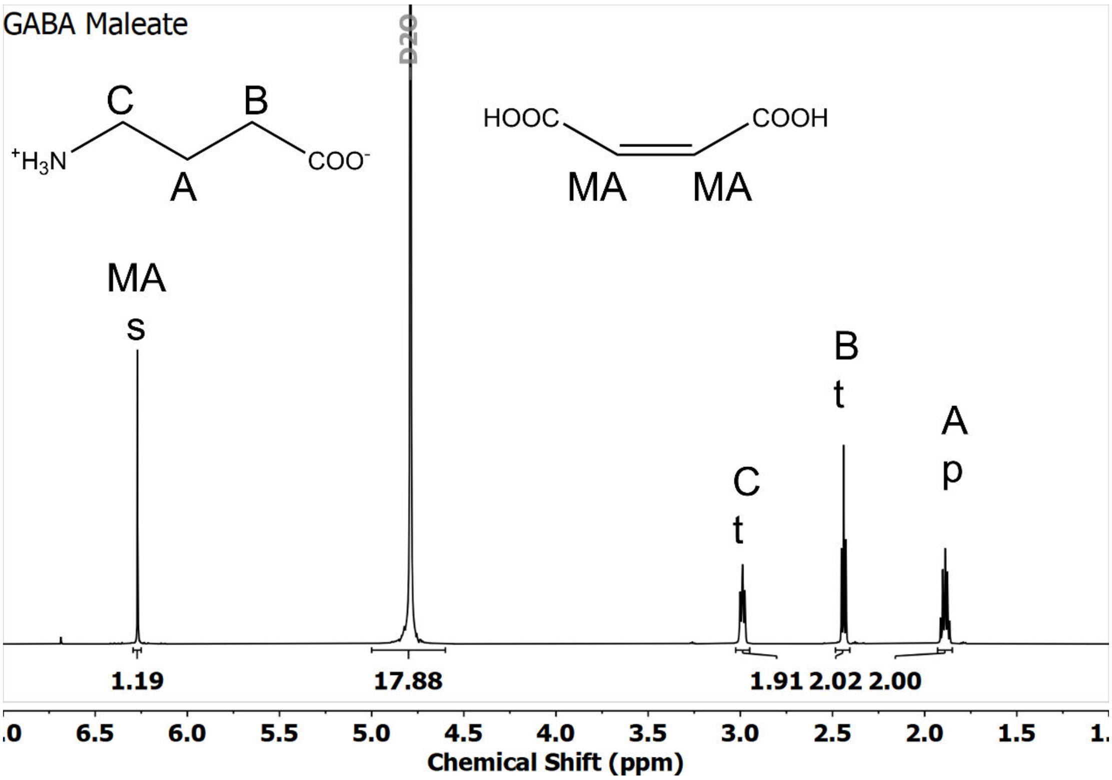

Figure S24. *^{1}H-NMR spectrum of GABA maleate recorded in D_2O at 600 MHz. Sample grown from solution.*

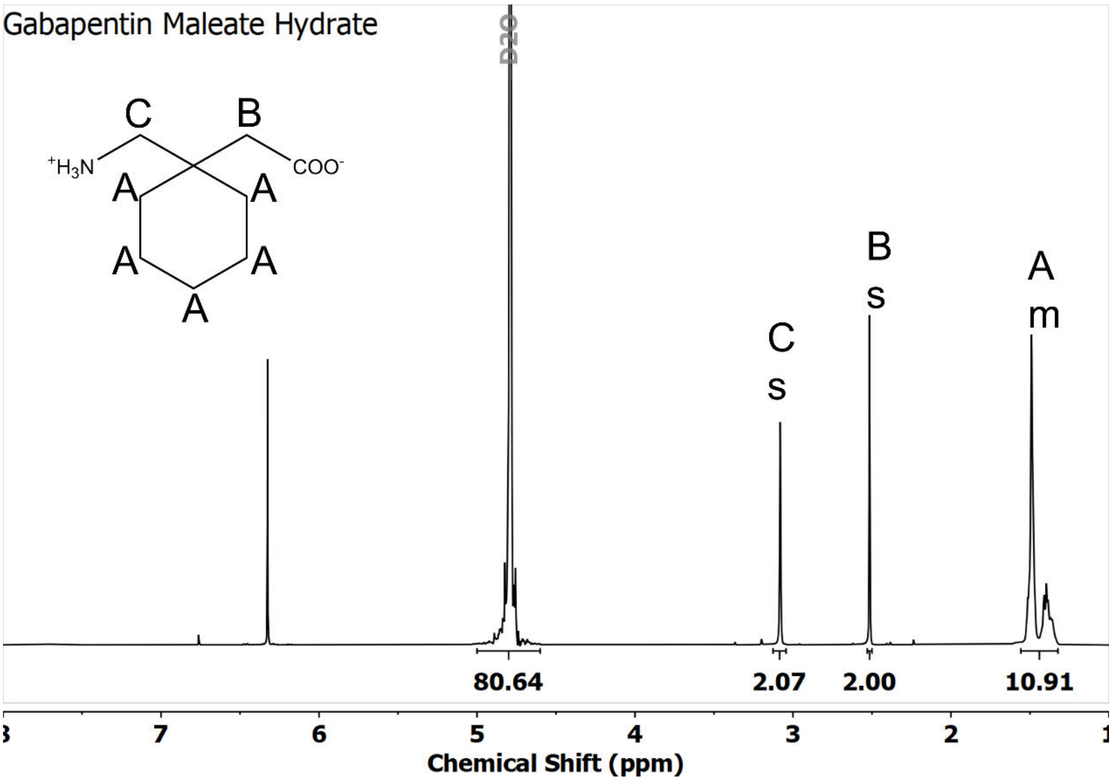

Figure S25. *^{1}H-NMR spectrum of Gabapentin maleate hydrate recorded in D_2O at 600 MHz.*

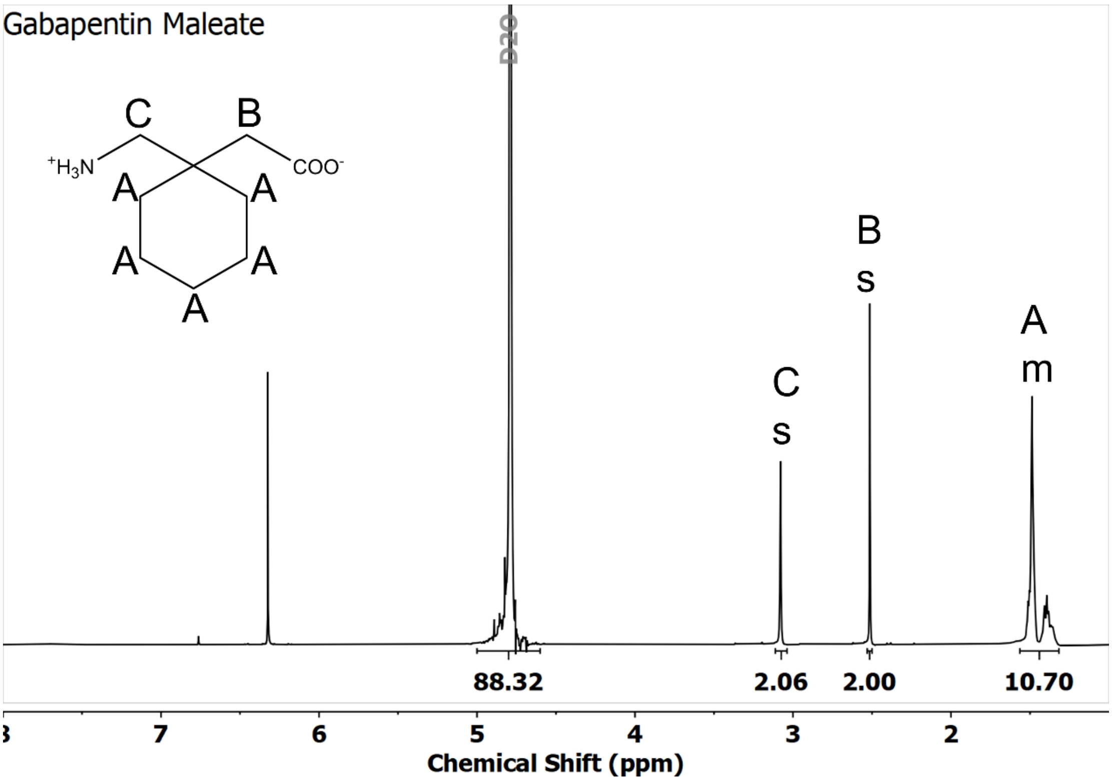

Figure S26. *^{1}H-NMR spectrum of Gabapentin maleate recorded in D_2O at 600 MHz.*

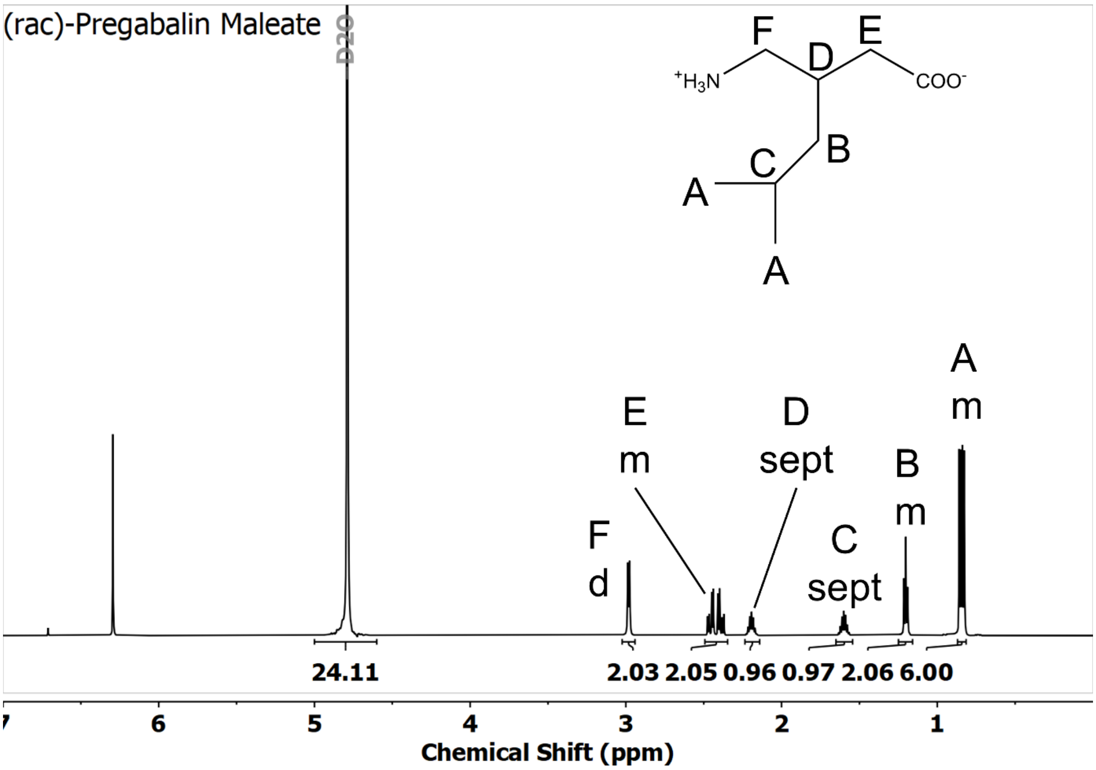

Figure S27. *^{1}H-NMR spectrum of (rac)-Pregabalin maleate recorded in D_2O at 600 MHz. Sample grown from solution.*

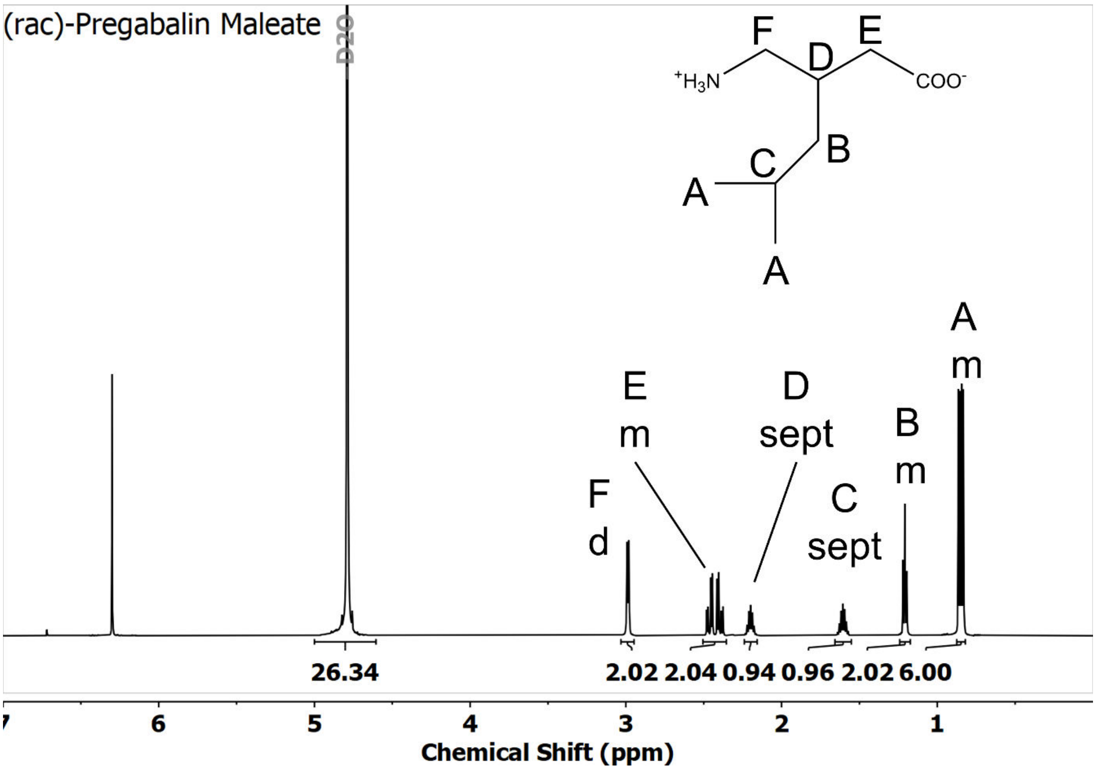

Figure S28. *^{1}H-NMR spectrum of (rac)-Pregabalin maleate recorded in D_2O at 600 MHz. Sample grown via grinding.*

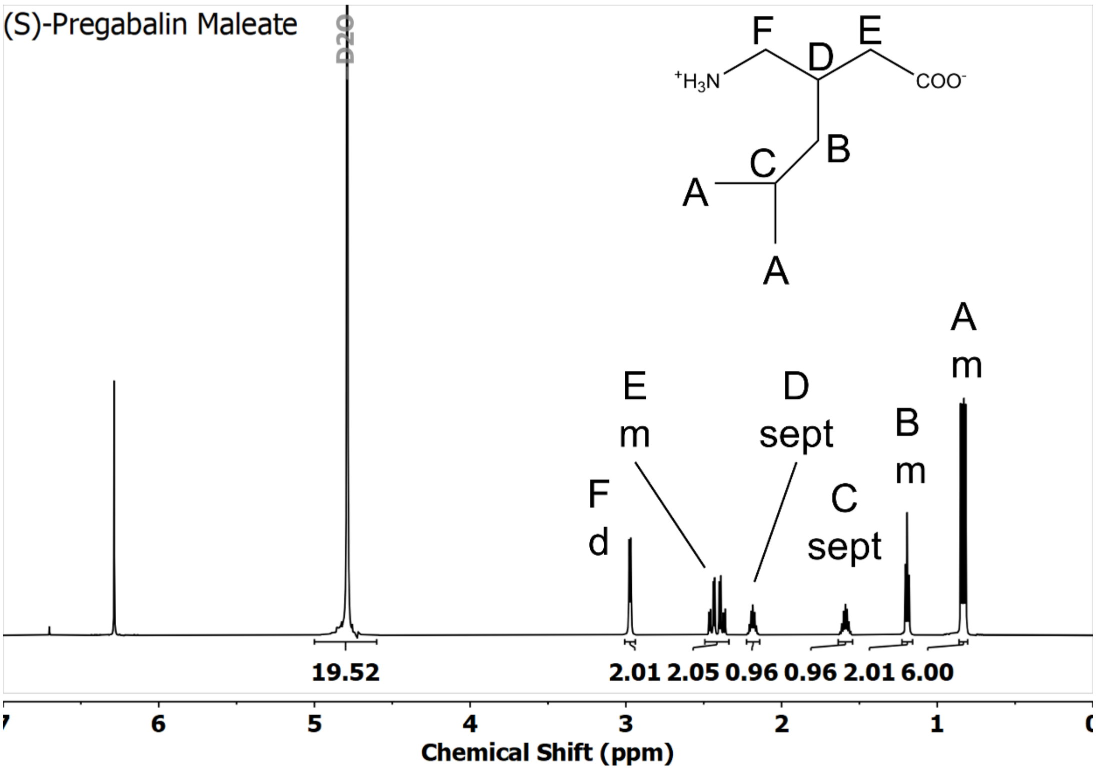

Figure S29. *^{1}H-NMR spectrum of (S)-Pregabalin maleate hydrate recorded in D_2O at 600 MHz. Sample grown from solution.*

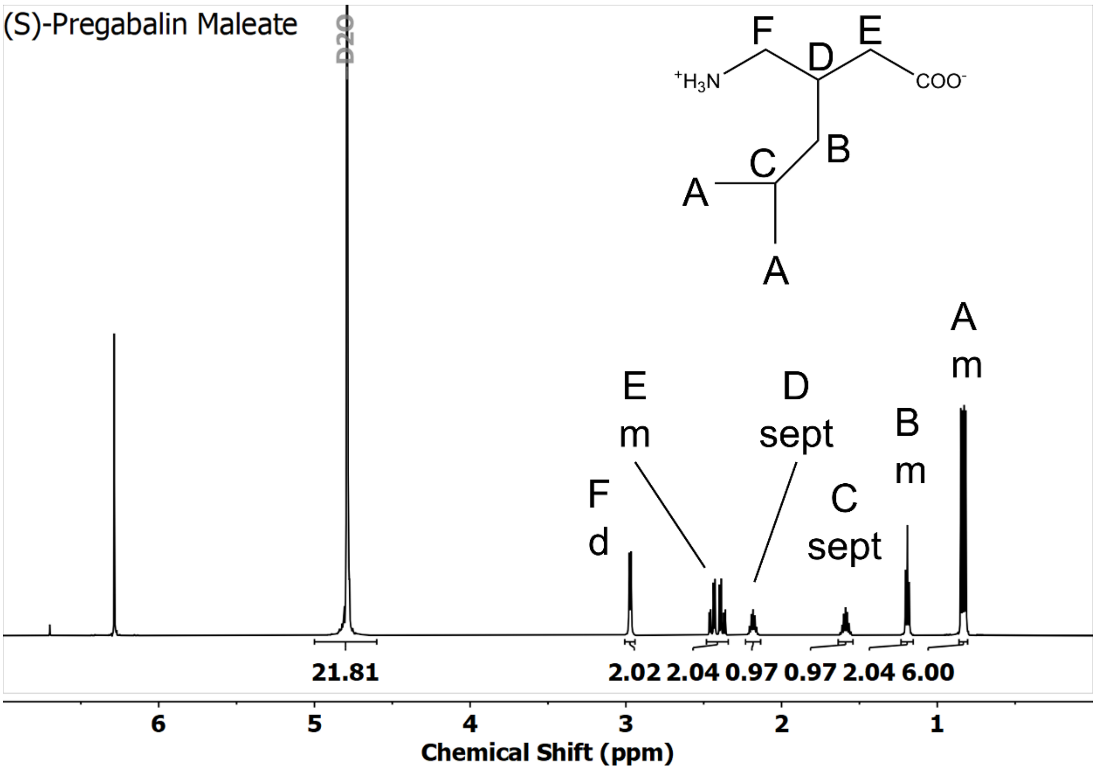

Figure S30. *^{1}H-NMR spectrum of (S)-Pregabalin maleate hydrate recorded in D_2O at 600 MHz. Sample grown via grinding.*

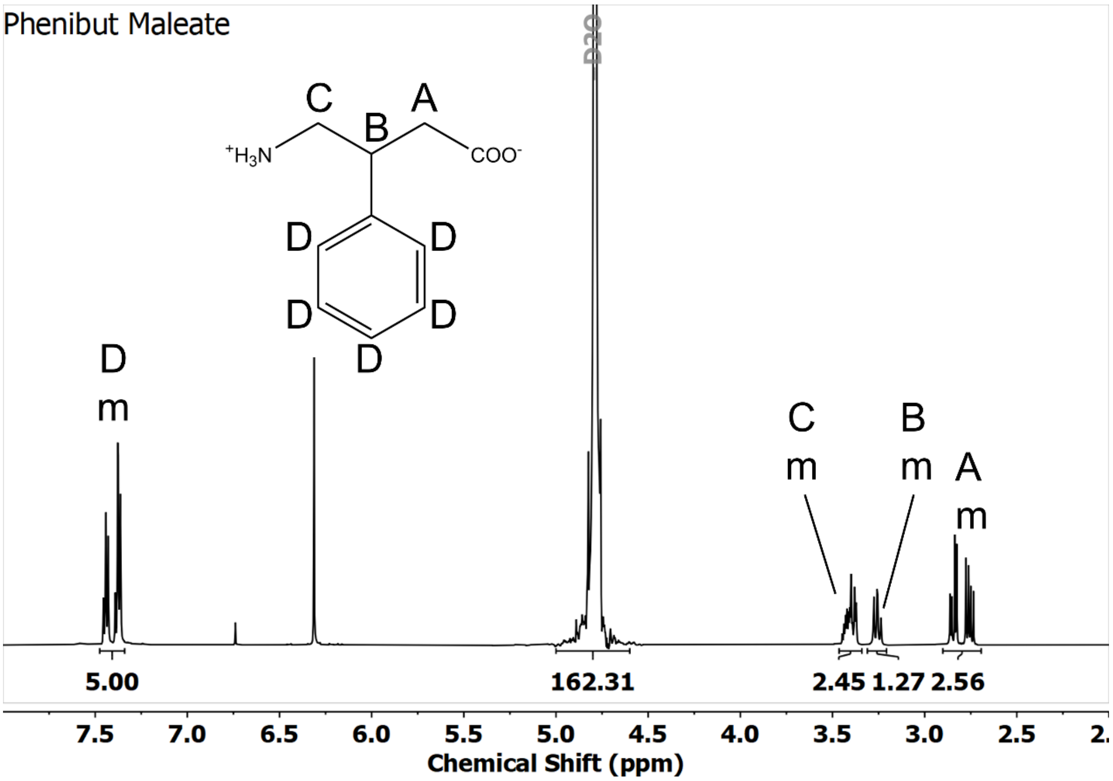

Figure S31. *^{1}H-NMR spectrum of Phenibut maleate recorded in D_2O at 600 MHz. Sample grown from solution.*

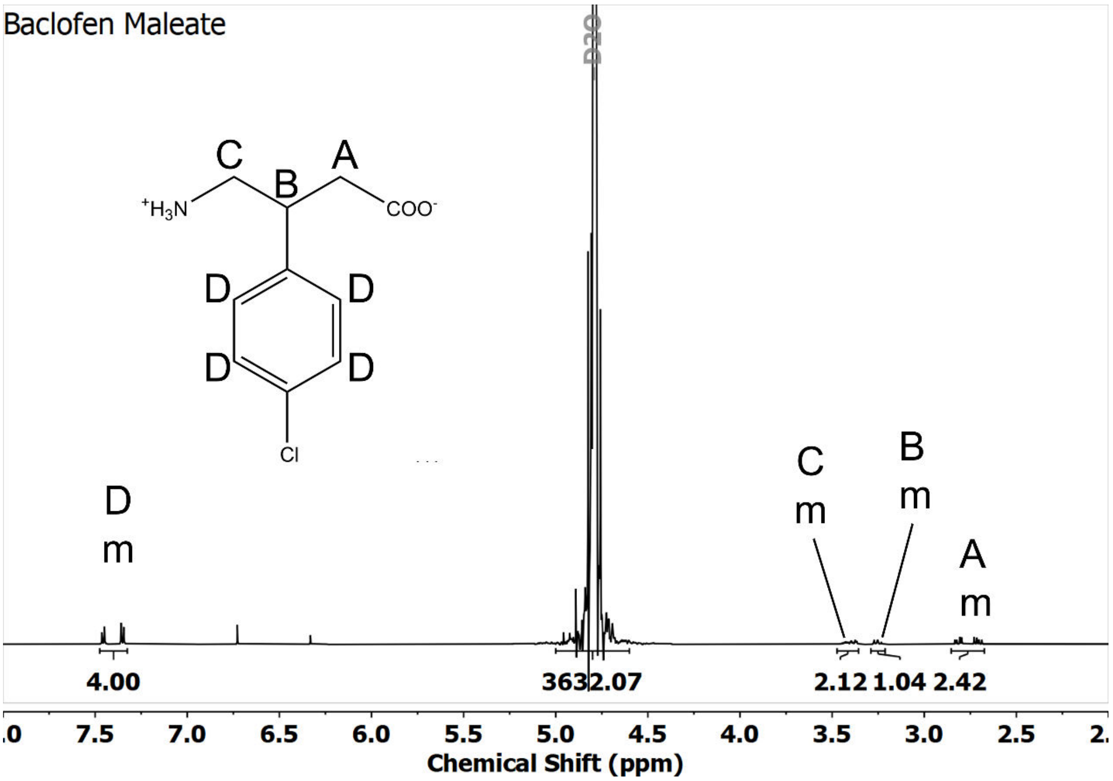

Figure S32. *^{1}H-NMR spectrum of Baclofen maleate recorded in D_2O at 600 MHz. Sample grown from solution.*
